# In Silico Modeling of Nanoparticle Transport across the Blood–Brain Barrier: A Systematic Review

**DOI:** 10.34133/csbj.0020

**Published:** 2026-03-25

**Authors:** Qianqian Xia, S. H. B. Herath Mudiyanselage, Sébastien Lafond, Hergys Rexha

**Affiliations:** ^1^ Faculty of Science and Engineering, Information Technology, Åbo Akademi University, Turku 20500, Finland.; ^2^ ESIGELEC Graduate School of Engineering, ISYMED (Medical Systems Engineering), Rouen 76000, France.

## Abstract

Reliable prediction of nanoparticle (NP) transport across the blood–brain barrier (BBB) is essential for designing effective central nervous system-targeted drug delivery systems. The BBB protects the brain but severely restricts the entry of therapeutic compounds, and fewer than 5% of candidate drugs reach the brain in pharmacologically meaningful amounts. NP-based delivery systems have emerged as a promising approach to overcome this limitation by enhancing drug stability, circulation, and BBB penetration. Experimental in vivo animal models and in vitro BBB assays provide valuable mechanistic insights but are costly, time-consuming, and limited in translational efficiency. In silico methodologies offer a complementary strategy by enabling efficient screening of NP designs, supporting interpretation of experimental data, and reducing dependence on animal models. This paper presents a systematic review of 56 peer-reviewed publications that applied computational methods to study NP transport across the BBB. The included works fall into 5 main categories: (a) molecular simulations, (b) quantitative structure–activity/property relationship models, (c) machine learning and deep learning approaches, (d) pharmacokinetic and pharmacodynamic modeling, and (e) nanoinformatics frameworks. These approaches address key stages of NP transport, including protein corona formation, interactions with endothelial membranes, and translocation across the barrier. By comparing these diverse methods, this review highlights their complementary strengths and integration potential for improving permeability prediction. Together, they demonstrate how multiscale computational modeling enhances understanding of NP behavior at the BBB, supports more ethical and nonanimal research, and paves the way for artificial intelligence-guided design of brain-targeted nanomedicines.

## Graphical Abstract

**Figure:**
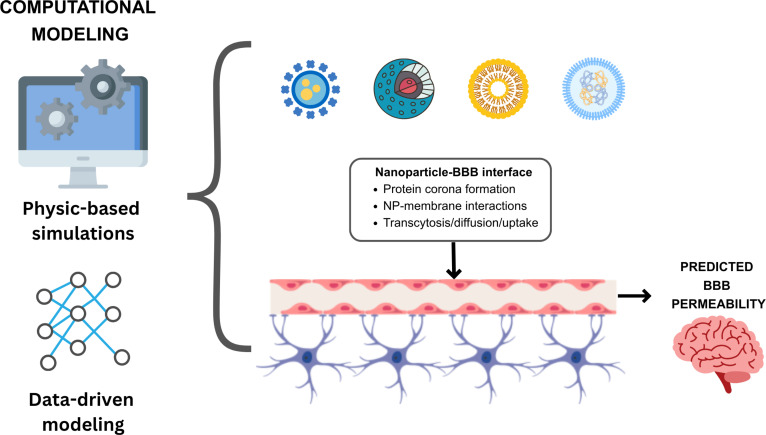

## Introduction

Brain diseases such as Parkinson’s, Alzheimer’s, Huntington’s, schizophrenia, dementia, and brain tumors affect more than 179 million Europeans and over one billion people worldwide [1,2]. They account for about €800 billion of healthcare costs in the European Union every year [3]. Effective treatment of these disorders remains challenging because the blood–brain barrier (BBB) protects the central nervous system (CNS) but prevents most therapeutic compounds from reaching the brain [4]. Nanoparticle (NP)-based drug delivery systems have emerged as promising carriers for crossing the BBB. Their modular architectures, tunable physicochemical features, and versatile ligand functionalization enable the rational design of therapeutics with enhanced BBB penetration [5,6]. Experimental and mechanistic studies have shown that NPs can traverse the BBB through multiple pathways, including paracellular diffusion, passive transcellular transport, carrier-mediated transport (CMT) via nutrient or ion transporters, and vesicular transcytosis mechanisms such as receptor-mediated transcytosis (RMT) and adsorptive-mediated transcytosis (AMT) [7,8]. Utilizing these physiological routes, various ligand-functionalized and surface-engineered NPs have improved BBB penetration and brain-targeted delivery [9].

Current in vitro and in vivo models provide valuable insights into BBB transport but remain limited in scalability, cost, and translatability. For example, tight junctions in endothelial cells restrict paracellular passage to only a few nanometers, making passive diffusion of NPs rare [10]. BBB-on-chip systems and animal studies have explained mechanisms such as receptor-mediated uptake and efflux transport by ATP-binding cassette transporters [7]. However, these experimental platforms cannot fully capture how physicochemical features of NPs, such as size, shape, surface charge, functionalization, and protein corona composition, interact with the dynamic BBB microenvironment [11–13]. As a result, despite over 3 decades of research, only about 30 NP formulations have progressed to clinical trials, with most failing at early stages due to limited BBB penetration, safety concerns, or poor translational predictivity [14]. These limitations highlight the need for quantitative, mechanistic, and scalable approaches that can complement experimental studies and reduce reliance on animal testing.

To overcome these experimental limitations, in silico modeling has emerged as a complementary, nonanimal strategy that enables mechanistic understanding and quantitative prediction of BBB transport processes. Recent improvements in computational power, simulation methodologies, and data-driven modeling have made it possible to investigate NP–BBB interactions at multiple scales, offering insights that are difficult to obtain through experimental approaches. However, existing reviews on computational modeling of NP transport across the BBB remain fragmented and limited in scope. Earlier works primarily examined small-molecule permeability or transporter-mediated mechanisms, typically employing structure–activity relationship (SAR) analyses and early machine learning (ML) frameworks [15]. Subsequent works have discussed NP–BBB interactions from specific perspectives, including nanotoxicity modeling [16], multiscale simulations in cancer nanomedicine [17], and physiologically based pharmacokinetic (PBPK) modeling of nanocarriers [18], but these remain narrow in scope and lack systematic integration across modeling scales. More recently, Singh et al. [19] emphasized AI-assisted nanorobotic design. Collectively, these efforts demonstrate a growing interest in computational approaches but remain limited either in scope (small-molecule or drug compound), depth (narrow methodological coverage), or transparency (absence of systematic evaluation).

This study addresses this gap by conducting a systematic literature review of in silico approaches for NP transport across the BBB. These approaches include computational frameworks capable of simulating or predicting NP–BBB interactions and transport phenomena across relevant biological scales. We consider the physiological mechanisms that govern these processes to ensure that the reviewed computational strategies remain closely aligned with experimentally observed behaviors. Structured data extraction and quality assessment are performed to evaluate their methodological contributions, limitations, and complementarities. The aim is to provide a transparent and structured overview of computational modeling frameworks representing NP transport across the BBB and to identify methodological gaps that motivate further integrative analysis.

The remainder of this paper is organized as follows. The “Methods” section describes the systematic search strategy and data extraction protocol following the PRISMA 2020 guidelines. The “Methodological Taxonomy of In Silico Approaches” section presents a taxonomy of computational approaches across modeling domains and biological scales. The “Cross-Scale Comparative Analysis of In Silico Approaches” section compares how existing approaches represent NP and BBB components, their supporting datasets, and their modeling scales. The “Evaluation and Limitations” section evaluates the methodological rigor and predictive performance and identifies limitations. Finally, the “Discussion” and “Conclusion” sections discuss the general implications and future work of the research, and present the conclusions, respectively.

## Methods

This section describes the methodological framework of the systematic review, including the formulation of research questions, search strategy, inclusion and exclusion criteria, screening process, overall information of the included studies, and quality assessment of the included studies.

### Research question

Following Kitchenham’s guidelines for systematic literature reviews [20], this work formulates research questions to systematically analyze how in silico approaches, defined as computational methods that model, simulate, or predict NP interactions with the BBB, have been applied to characterize and predict NP penetration across the BBB, with particular attention to their methodological diversity, strengths, limitations, and research gaps. Building upon the motivation and objectives outlined in the “Introduction” section, this review addresses 5 research questions (RQ1 to RQ5), as summarized in Table 1.

**Table 1.** Research questions and corresponding sections addressing them

RQ	Research question	Addressed in section
RQ1	How can NPs’ physicochemical properties and their biological transport environments be represented in silico?	“Biological mechanism and analytical framework” and “Feature representations”
RQ2	How have in silico approaches been employed to investigate key biological processes underlying NP–BBB interactions?	“Distribution of modeling approaches” and “Modeling resolution and computational scales”
RQ3	How can results from multiscale simulations and experimental datasets be integrated to develop predictive models for NP permeability and BBB crossing efficiency?	“Hierarchical taxonomy structure” and “Key datasets and reuse”
RQ4	How are existing in silico models assessed for methodological completeness, validation, and reproducibility, and what limitations and biases can be identified?	“Model performance and validation strategies” and “Limitations”
RQ5	What future directions and AI-assisted strategies can enhance the integration, interpretability, and predictive power of computational modeling frameworks for NP–BBB interactions?	“Model performance and validation strategies” and “Future work”

AI, artificial intelligence; BBB, blood–brain barrier; NP, nanoparticle

### Search methodology

This systematic literature review was conducted following the Cochrane Handbook and the most up-to-date Preferred Reporting Items for Systematic Reviews and Meta-Analyses (PRISMA) 2020 guidelines [21]. The methodology included a structured search strategy, screening process, and data extraction protocol with quality assessment and scoring of methodological rigor, data transparency, and validation practices across all included studies.

A systematic literature search was conducted in May 2025 using PubMed, Scopus, and Web of Science. The search strategy was built around 3 groups of keywords (P1 to P3) combined with Boolean logic to capture the main aspects of the topic: (P1) NP context, (P2) BBB and transport mechanisms, and (P3) in silico and computational methods. Within each group, related keywords, synonyms, and acronyms were connected with the operator OR, and the complete query was formulated as P1 AND P2 AND P3. NP-related terms (P1) covered diverse NP types and nanocarrier descriptors to ensure a broad representation of delivery vehicles. BBB and transport terms (P2) were required to appear explicitly in the article title to ensure central relevance to BBB research, encompassing not only “blood–brain barrier”, “BBB transport”, “permeability”, and “transcytosis”, but also mechanism-specific and biological interaction terms such as “receptor-mediated transport”, “adsorptive-mediated transport”, “endocytosis”, “protein corona formation”, “cellular association”, and “uptake”. Computational and modeling terms (P3) focused on simulation- and data-driven methodologies such as molecular dynamics simulation, modeling, machine learning, and deep learning (DL), ensuring a primary computational emphasis. To improve precision, BBB- and modeling-related terms (P2 and P3) were searched only within the title field, whereas NP-related terms (P1) were searched within both the title and abstract fields. All searches were limited to English-language journal articles, as summarized in Table 2, with full search strings provided in the Supplementary Materials (SM1).

**Table 2.** Summary of database search queries and number of records retrieved

No.	Database	Search query (simplified expression)	Papers found
1	PubMed	([P1:Title/Abstract]) AND ([P2:Title]) AND ([P3:Title])	119
2	Scopus	(TITLE-ABS-KEY(P1)) AND (TITLE(P2)) AND (TITLE(P3))	145
3	Web of Science	TS=(P1) AND TI=(P2) AND TI=(P3)	407

### Inclusion and exclusion criteria

This systematic review aimed to identify computational approaches to model and evaluate NP transport across the BBB, with emphasis on in silico methods providing mechanistic or predictive insights into NPs permeability. The inclusion criteria were defined with 2 main considerations: methodological rigor and scientific relevance. From a methodological perspective, the review focused on studies employing in silico approaches, rather than isolated or narrowly defined wet-lab experiments. From a scientific standpoint, the studies need to address processes relevant to brain-targeted nanodrug delivery or CNS, including NP–membrane interactions, translocation mechanisms, and other phenomena contributing to BBB permeability.

Based on these considerations, a set of inclusion and exclusion criteria was established to ensure that only original, high-quality, and computationally grounded research was analyzed. The detailed criteria are summarized in Table 3.

**Table 3.** Inclusion and exclusion criteria

Code	Criterion description
Inclusion criteria (IC)	
IC1	Original research articles published in peer-reviewed journals.
IC2	Articles written in English with full text available.
IC3	Studies employing computational (in silico) approaches, including simulation, statistically modeling, or dataset analysis, to investigate NP interactions with the BBB as a biological system.
IC4	Studies focusing on NP transport across the BBB, with an explicit NP system, and on mechanistically related processes (protein corona formation, NP–membrane interaction, endocytosis, exocytosis, intracellular trafficking, or diffusion).
IC5	Studies providing mechanistic or predictive insights into NP permeability relevant to CNS drug delivery.
Exclusion criteria (EC)	
EC1	Reviews, systematic reviews, meta-analyses, books, or book chapters.
EC2	Conference abstracts without full-text articles.
EC3	Studies based solely on in vivo or in vitro experiments without computational components.
EC4	Studies addressing diseases unrelated to the CNS (e.g., breast, lung, or skin models).
EC5	Purely theoretical or mathematical papers lacking implementation, code, or simulation results.

BBB, blood–brain barrier; CNS, central nervous system; NP, nanoparticle

### Study selection procedure

**Fig. 1.**
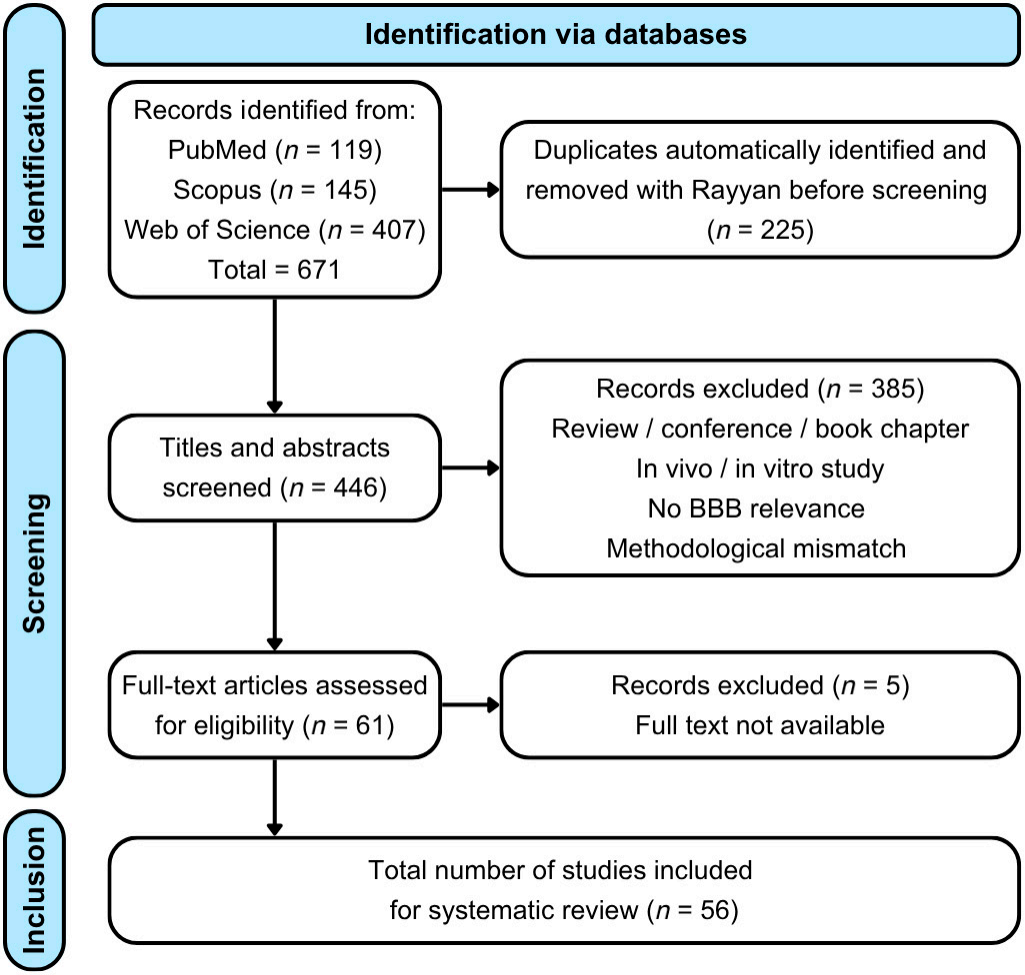
PRISMA 2020 flow diagram of the study selection process. A total of 671 records were identified from Web of Science, Scopus, and PubMed. After removing 225 duplicates automatically with Rayyan, 446 titles and abstracts were screened. A total of 61 full-text articles were assessed for eligibility, and 5 were excluded because the full text was not available. In total, 56 studies were included in the final systematic review.

All retrieved records were first imported into Rayyan [22] for automatic duplicate removal and subsequent manual screening. The first 2 authors independently screened the titles and abstracts in Rayyan according to the predefined inclusion and exclusion criteria, without viewing each other’s decisions. Each reviewer labeled studies as “include”, “exclude”, or “uncertain”. The system then identified conflicts, which were jointly discussed to reach a consensus by the first 2 authors. After this stage, the screened records were imported into Zotero for full-text management. Articles without accessible full text were removed at this step. The final set of eligible full-text studies, organized in Zotero, formed the basis for data extraction and analysis. The overall selection process followed the PRISMA 2020 workflow and is summarized in Fig. 1.

### Overview of included studies

**Fig. 2.**
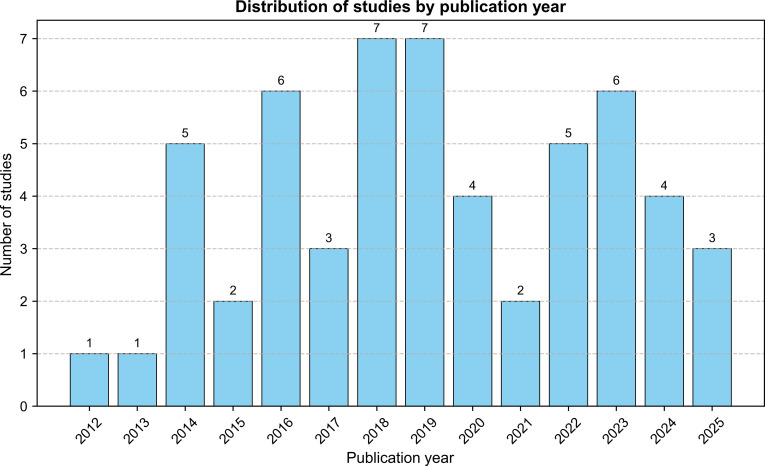
Distribution of studies by publication year (2012 to 2025).

**Fig. 3.**
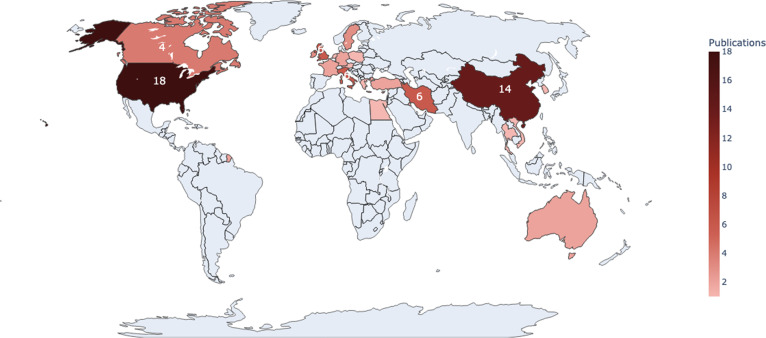
Global distribution of included studies by country. Darker colors represent a higher number of publications.

A total of 56 original research articles published between 2012 and 2025 were included in this review. As shown in Fig. 2, publication activity increased notably after 2014, peaking in 2018 and 2019 with 7 studies each, and showing renewed growth between 2022 and 2024, highlighting the sustained interest in applying computational methods in this domain. Most studies originated from the United States (18), China (14), the United Kingdom (6), Italy (6), Iran (6), Canada (4), Ireland (3), and Sweden (3). Because some studies involved international collaborations, each contributing country was counted separately. This approach shows the global distribution of research in this field, as shown in Fig. 3.

### Quality assessment and reproducibility

To enable systematic quality assessment and reproducibility analysis, we extracted key methodological information from all 56 included studies. For each study, we first collected general bibliographic information (e.g., authors, year, digital object identifier, and institution) alongside detailed methodological descriptors. For studies involving molecular simulations, we collected details on the simulation type, force field, software, scale, and duration, as well as how NP physicochemical characteristics (size, shape, core, and surface chemistry) and the composition of the BBB model were simulated. We also tracked key outputs such as interaction energies, protein corona features, and validation methods. For studies applying statistical or ML analysis to datasets, we captured key aspects of data sources, input descriptors, labeling strategies, algorithm types, and model performance metrics.

To ensure methodological rigor, transparency, and reproducibility, each included study was evaluated according to 3 assessment criteria summarized in Table 4. These criteria were designed to systematically quantify key aspects of model quality and reporting: Model Simplification Bias (AC1), Data Availability Bias (AC2), and Validation Bias (AC3). Each study received a score between 0 and 2 for each criterion, with higher scores reflecting greater methodological transparency and reproducibility. An overall average score was then calculated to represent the general quality level of each study. The detailed scoring metrics and examples for AC1 to AC3 are provided in the Supplementary Material (SM3), and the complete scoring outcomes for all included studies are summarized in the Supplementary Material (SM4).

**Table 4.** Assessment criteria (AC1 to AC3) for evaluating study quality and reproducibility

Code	Criterion description
AC1	Model simplification bias: Evaluates how completely and realistically the model represents NP transport across the BBB. • It considers whether the study captures relevant BBB transport stages defined in this review. • It also examines whether BBB-specific features such as endothelial cells, tight junctions, membrane composition, or receptors are represented to reflect realistic in vivo or in vitro conditions.
AC2	Data availability bias: Evaluates the transparency, documentation, and accessibility of the data and computational artifacts that enable reproducibility and independent verification. • For simulation work, it evaluates the completeness and openness of simulation inputs, force fields, configuration files, and trajectory data, as well as the availability of analysis scripts and metadata [102,103]. • For data-driven and machine learning work, it evaluates the accessibility and documentation of datasets, preprocessing methods, and model implementation details, following open-science and FAIR (Findable, Accessible, Interoperable, and Reusable) principles [104].
AC3	Validation bias: Evaluates the rigor, transparency, and reproducibility of model validation procedures. • For simulation work, it evaluates whether results are quantitatively or qualitatively compared with experimental data, literature benchmark systems, or other simulation scales to ensure physical consistency [28,105]. • For data-driven and machine learning work, it evaluates the use of systematic validation strategies such as cross-validation, independent test or external datasets, and performance reporting, as well as any integration with wet-lab experimental validation to confirm model predictivity and generalizability [104].

BBB, blood–brain barrier; NP, nanoparticle

## Methodological Taxonomy of In Silico Approaches

This section outlines the biological mechanism in 3 stages and introduces a hierarchical taxonomy organizing studies by modeling domain and scale, methodological type, and mechanistic stage, providing a unified framework to compare in silico approaches.

### Biological mechanism and analytical framework

**Fig. 4.**
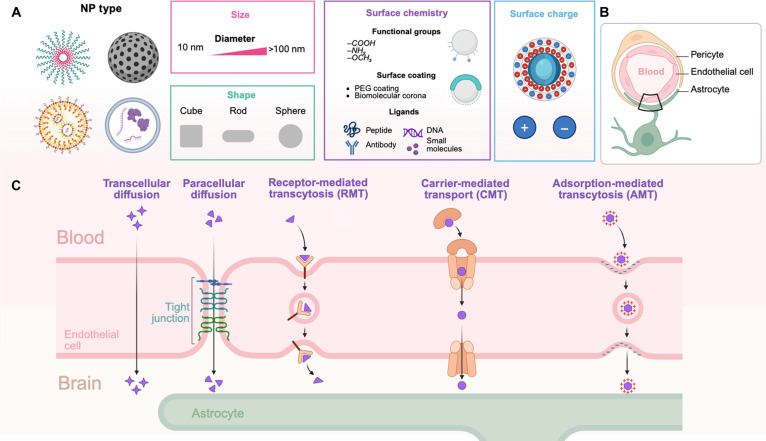
Overview of the blood–brain barrier (BBB) and NP interaction mechanisms. (A) Key physicochemical properties of nanoparticles, including size, shape, surface chemistry, and charge, that influence BBB permeability. (B) Structural components of the BBB and neurovascular unit (NVU), including endothelial cells, pericytes, and astrocytes. (C) Principal transport pathways across the BBB: passive diffusion (transcellular and paracellular) and active mechanisms including receptor-mediated transcytosis (RMT), carrier-mediated transport (CMT), and adsorption-mediated transcytosis (AMT). Figure created with BioRender.com.

Before analyzing computational approaches, it is essential to understand the physiological mechanisms that govern NP transport across the BBB, as these form the biological foundation for all subsequent modeling efforts. The BBB is a dynamic interface composed mainly of brain endothelial cells connected by tight junctions and supported by pericytes, astrocytes, and the basement membrane [12,13]. Together, these components form the neurovascular unit (NVU) that regulates molecular exchange between blood and brain, as illustrated in Fig. 4B.

The BBB excludes more than 98% of small-molecule drugs and all macromolecular therapeutics from accessing the brain at pharmacologically relevant concentrations [11]. As illustrated in Fig. 4C, small lipid- or water-soluble molecules can passively cross via paracellular or transcellular diffusion. However, this pathway is usually not available for most NPs, because the tight junctions are extremely small (from 4 to 6 nm). Only ultrasmall NPs, such as gold NPs of about 1 to 2 nm, have occasionally been observed to passively cross the BBB under special conditions [10]. NPs may instead utilize several active or facilitated mechanisms, including RMT, CMT, and adsorption-mediated transcytosis (AMT). In RMT, endothelial receptors such as transferrin (TfR), insulin (IR), and low-density lipoprotein receptors actively shuttle ligand-functionalized NPs across the BBB [8,9,23]. For example, lactoferrin-conjugated PEGylated Fe_3_O_4_ NPs have demonstrated enhanced BBB penetration via RMT [8]. CMT utilizes solute carrier proteins like GLUT1 and LAT1 that transport glucose and amino acids; NPs functionalized with similar moieties can exploit these transporters to improve uptake [7]. AMT involves electrostatic interactions between cationic NPs and negatively charged endothelial membranes, providing a nonspecific yet efficient route for BBB translocation [24,25].

In contrast, ABC efflux pumps such as P-gp and BCRP expel xenobiotics from endothelial cells, limiting brain accumulation [7]. While physical or biochemical disruption methods (e.g., focused ultrasound or osmotic agents) can temporarily increase BBB permeability [23,25], this review focuses on transport under normal physiological conditions.

Overall, NPs may use multiple routes to reach the brain, such as passive diffusion, transcytosis, or active transport. Their permeability is strongly influenced by physicochemical design parameters such as size, shape, surface charge, and surface chemistry, including ligand functionalization, which modulate NP interactions with the biological environment [25,26]. As illustrated in Fig. 4A, these factors are commonly investigated through in silico models to elucidate mechanistic contributions and to serve as predictive inputs for modeling BBB-related transport behavior. In addition, once introduced into the bloodstream, NPs rapidly interact with plasma proteins and other biomolecules, forming a dynamic biomolecular (protein) corona that governs their pharmacokinetics, biodistribution, and cellular interactions [27], thereby affecting subsequent transport across the BBB. Building on this mechanistic understanding, in silico modeling offers an effective means to simulate and predict NP-BBB interactions across multiple transport stages, providing complementary insights into NP physicochemical properties, protein adsorption dynamics, and BBB membrane binding. In this review, we define these stages as (a) protein corona formation, (b) NP–membrane interactions, and (c) cross-BBB passage, which serve as the analytical framework for evaluating the included studies, as summarized in Table 5.

**Table 5.** Mechanistic stages of nanoparticles transport across the BBB

Stage	Description
Protein corona formation	In blood, NPs rapidly adsorb biomolecules such as proteins, lipids, and metabolites, forming a biomolecular corona that defines their biological identity. The protein-rich fraction, known as the protein corona, primarily governs NP pharmacokinetics, biodistribution, and recognition by cells and the immune system.
NP–membrane interactions	Corona-coated NPs reach the BBB endothelium and interact with the luminal membrane via electrostatic attraction, ligand–receptor binding (e.g., transferrin, insulin, and LDL), or partial membrane embedding. These interactions initiate cellular uptake and transcytosis.
Cross-BBB transport	Internalized NPs traverse the endothelium through receptor-, adsorptive-, or carrier-mediated transcytosis, or limited passive diffusion. Vesicular trafficking delivers them to the abluminal side, enabling entry into brain tissue for CNS targeting.

BBB, blood–brain barrier; CNS, central nervous system; LDL, low-density lipoprotein; NP, nanoparticle

### Hierarchical taxonomy structure

**Fig. 5.**
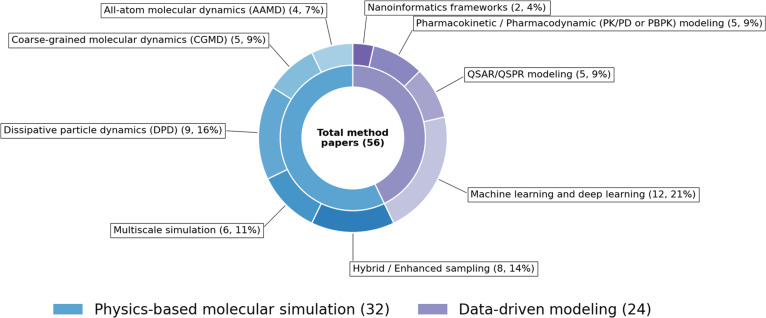
Distribution of computational methods across the 56 included studies. The inner ring represents 2 main paradigms: physics-based molecular simulations (blue, 32 studies, 57%) and data-driven modeling (purple, 24 studies, 43%). The outer ring shows subcategories including AAMD, CGMD, dissipative particle dynamics (DPD), hybrid/enhanced sampling, and multiscale simulations on the simulation side, and machine learning/deep learning (ML/DL), quantitative structure–activity/property relationship (QSAR/QSPR), pharmacokinetic/pharmacodynamic (PK/PD), or physiologically based pharmacokinetic (PBPK), and nanoinformatics approaches on the data-driven side. The distribution indicates a relatively balanced landscape between physics-based and data-driven approaches, reflecting the complementary roles of mechanistic simulations and predictive modeling in NP–BBB research.

In the context of NP transport across the BBB, 2 complementary computational paradigms have evolved. Physics-based molecular simulations provide atomistic and coarse-grained insights into NPs and biological interactions, while data-driven modeling approaches integrate experimental and simulation data to build predictive models. As illustrated in Fig. 5, 56 reviewed studies show a balanced distribution between simulation-based and data-driven approaches. Based on their modeling objectives and biological scales, we identified 5 major methodological categories: (a) molecular-level simulations including all-atom molecular dynamics (AAMD), coarse-grained molecular dynamics (CGMD), dissipative particle dynamics (DPD), multiscale simulations, and hybrid or enhanced sampling techniques; (b) descriptor-based quantitative models, primarily quantitative structure–activity/property relationship (QSAR/QSPR) approaches; (c) data-driven AI models, including ML and DL frameworks; (d) system-level kinetic models such as pharmacokinetic (PK) and pharmacodynamic (PD) models and PBPK models; and (e) nanoinformatics frameworks, encompassing specialized databases, ontologies, and workflow systems.

We propose to organize the 5 modeling domains into a concise 3-level taxonomy that links (a) domains and their representation scales, (b) specific computational methods, and (c) mechanistic stages of NP–BBB transport (Table 6). This hierarchical framework unifies previously disparate computational efforts, resolves the longstanding fragmentation of in silico approaches in the field, and provides a structured foundation for the rational development of accurate, scalable, and interoperable predictive models for NP transport across the BBB.

**Table 6.** Hierarchical taxonomy of computational approaches for NP–BBB modeling

Modeling domain	Representative methods	Mechanistic focus	Typical objectives
Molecular simulations	All-atom MD, coarse-grained MD, DPD, multiscale, hybrid and hybrid/enhanced-sampling simulations	Stage 1: Protein corona formation; Stage 2: NP–membrane interactions; Stage 3: RMT or AMT guided passage	Quantify adsorption energies, free-energy barriers, adhesion and wrapping dynamics; parameterize higher-scale models for uptake and permeability prediction.
Descriptor-based quantitative models	QSAR/QSPR	Stage 1: Protein corona formation; Stage 2–3: NP–BBB interactions and cellular uptake	Correlate NP physicochemical descriptors (size, charge, and surface chemistry) with measured association or uptake data.
Data-driven AI models	Random Forest, SVM, XGBoost, LightGBM, CNN	Stage 1: Protein corona profiling and formation; Stage 2–3: NP–BBB interactions and cellular uptake	Capture nonlinear and high-dimensional patterns from multisource datasets (experimental, omics, simulation) to predict corona composition or uptake rates.
System-level kinetic models	Compartmental PBPK, nonlinear fitting, ODE-based simulations	Stage 3: Tissue distribution (brain–plasma), systemic pharmacokinetics	Simulate time-dependent NPs concentrations across organs, translating permeability constants into in vivo biodistribution and exposure outcomes.
Nanoinformatics frameworks	FAIR-compliant databases (e.g., NanoCommons, eNanoMapper), ontologies, workflow automation	Cross-scale data integration (Stages 1–3)	Standardize metadata and modeling workflows; enable reproducible and interoperable NP–BBB simulation and prediction pipelines.

AMT, adsorptive-mediated transcytosis; CNN, convolutional neural network; DPD, dissipative particle dynamics; FAIR, Findable, Accessible, Interoperable, and Reusable; MD, molecular dynamics; ODE, ordinary differential equation; PBPK, physiologically based pharmacokinetic; QSAR/QSPR, quantitative structure–activity/property relationship; RMT, receptor-mediated transcytosis; SVM, support vector machine; XGBoost, extreme gradient boosting

### Distribution of modeling approaches

The following subsections summarize representative works within each methodological categorization.

#### Molecular-level simulations

Molecular-level simulations represent the largest category in this review (32 of 56 studies) and include AAMD, CGMD, DPD, multiscale, and hybrid/enhanced-sampling approaches as summarized in Table 7). These techniques function as a “computational microscope”, revealing biomolecular events at spatiotemporal scales inaccessible to current experiments [28].

**Table 7.** Representative physics-based simulation approaches for NP–BBB modeling, highlighting typical applications and computational tools

Modeling approach	Applications	Main tools and frameworks
All-atom molecular dynamics (AAMD) [81–84]	Lipid-based nanoparticle interfaces [81]; charged or functionalized metal; peptide and ligand conformational behavior [83]; surface interactions [82]; polymer–drug encapsulation [84].	**Engines:** GROMACS, LAMMPS **Force Fields:** AMBER, OPLS-AA, GolP, PCFF, TIP4P **Analysis**: Umbrella sampling, WHAM analysis, VMD
Coarse-grained molecular dynamics (CGMD) [37,39,41,89,90]	Protein corona formation [41,89,90]; NP–membrane interactions[39]; receptor-mediated uptake[37].	**Engines:** GROMACS, LAMMPS, NAMD **Force fields:** CHARMM36/36m, MARTINI (2.0–3.0), Slipids, TIP3P water **Analysis:** PLUMED, WHAM, DSSP, APBS, VMD, Python scripts
Dissipative particle dynamics (DPD) [34–36,38,42,91–94]	Protein corona formation [42,91]; NP–membrane adhesion and wrapping [34,36,92,93]; receptor-mediated and cooperative endocytosis [35,38,94].	**Engines:** LAMMPS, custom DPD codes **Force fields:** Standard DPD bead–spring potentials with electrostatic and receptor–ligand extensions **Analysis:** VMD, OVITO, Python scripts
Multiscale modeling [77–79,85–87]	Protein corona restructuring [85–87]; NP adsorption energetics and orientation [77,79]; membrane wrapping and uptake [78].	**Engines:** GROMACS, LAMMPS **Force fields:** MARTINI 3.0, CHARMM36, AMBER03, Slipids, TIP3P water **Analysis:** PLUMED (metadynamics, umbrella sampling), DSSP, APBS, Python scripts
Hybrid/Enhanced sampling [40,95–100,106]	Protein corona and charge control [95,96]; NP–membrane adhesion and penetration [97]; umbrella sampling of NP uptake [40,98]; BBB transport under magnetic forces [99,100,106].	**Engines:** GROMACS, NAMD, LAMMPS Force Fields: CHARMM36/27, CGenFF, MARTINI (2.2–3.0), GolP, TIP3P/mTIP3P **Enhanced sampling:** Steered MD, Umbrella sampling, Well-tempered metadynamics, MDMCCE hybrid **Analysis:** PLUMED, WHAM, APBS, VMD, Python/MATLAB

BBB, blood–brain barrier; MD, molecular dynamics; NP, nanoparticle

Given the multicellular complexity of the BBB (see the “Biological mechanism and analytical framework” section), simulating complete NP translocation across the NVU remains computationally infeasible. Instead, due to computational constraints and spatial limitations, molecular simulations typically represent the BBB as a simplified, localized barrier segment to investigate molecular-level events [29–31]. For example, simple single-component bilayers such as 1,2-dioleoyl-sn-glycero-3-phosphocholine (DOPC) or 1-palmitoyl-2-oleoyl-sn-glycero-3-phosphocholine (POPC) are commonly used to study the passive solubility diffusion of small molecules [30,32], whereas mixed-lipid bilayers containing cholesterol and brain-relevant phospholipids offer a more accurate representation of the density and viscosity of the human brain microvascular endothelial cell (hBMEC) membrane [29,31,33]. However, these models, originally designed for solute permeation, fail to capture the complex transport physics of NPs. Unlike small molecules that diffuse through the free volume of the bilayer, NPs require marked membrane deformation or wrapping to enter the cell. This process is often driven by active machinery or specific ligand–receptor interactions that trigger endocytosis.

To better reflect biological transport mechanisms, more realistic membrane models now incorporate heterogeneous lipid compositions and specific transmembrane proteins. DPD and CGMD simulations reveal the competitive interplay between RMT and AMT. These methods provide insights that remain largely inaccessible to experimental approaches because they can quantify the distinct driving forces (ligand–receptor affinity for RMT versus electrostatic interactions for AMT) and the associated thermodynamic energy barriers. For RMT, several simulation studies have progressively expanded the mechanistic understanding of ligand–receptor-driven internalization. One DPD study examined the adhesion phase of ligand-tethered lipid–polymer hybrid NPs (LPH NPs), capturing the initial receptor-binding and membrane-deformation events but stopping before full wrapping or transcytosis [34]. DPD models have also investigated the influence of NP geometry, modeling spheres, rods, and disks interacting with membrane receptors and decomposing the internalization pathway into an invagination stage driven by ligand–receptor binding and a wrapping stage governed by membrane-bending energetics [35]. Other mesoscale DPD studies have introduced virus-like particles with tunable spike lengths and directly simulated receptor binding, receptor recruitment, and membrane budding, revealing a biphasic dependence of endocytosis efficiency on spike length [36]. CGMD simulations have likewise modeled ligand-decorated elastic NPs interacting with receptor-bearing membranes, capturing the full thermodynamics of membrane wrapping and quantifying how ligand–receptor affinity can overcome the bending-energy barrier required for successful internalization [37]. Regarding AMT, mesoscale simulations have examined how electrostatic interactions between charged NPs and negatively charged membranes initiate adsorptive uptake. One DPD study modeled positively charged NPs interacting with anionic lipid bilayers and revealed a cooperative endocytosis mechanism, in which membrane deformation induced by one NP facilitates the wrapping of neighboring particles, thereby overcoming electrostatic repulsion and promoting collective internalization [38]. Another simulation study investigated NPs with different surface chemistries interacting with mixed 1,2-dipalmitoyl-sn-glycero-3-phosphocholine (DPPC) / 1,2-dipalmitoyl-sn-glycero-3-phosphoglycerol (DPPG) membranes and demonstrated that positively charged surfaces (e.g., PEG^+^) rapidly bind to anionic lipids, inducing curvature and local membrane wrapping characteristic of AMT, whereas the presence of a protein corona can screen these electrostatic interactions and inhibit adsorption-driven uptake [39]. CGMD studies have also simulated the uptake of gold NPs with different shapes and surface charges into complex mammalian plasma membrane models, showing that cationic particles show faster membrane wrapping and internalization, whereas smaller neutral particles may translocate directly across the bilayer, collectively capturing the key mechanics of AMT and charge-driven translocation in a realistic membrane environment [40]. Furthermore, acknowledging that transport is often mediated by the NP’s biological identity, mesoscale simulations have examined how zwitterionic surface modifications minimize electrostatic interactions with serum proteins, thereby preserving the surface characteristics required to either enable or inhibit downstream RMT or AMT pathways [41]. Another study has compared hydrophobic, cationic, and ligand-functionalized NPs to evaluate how corona adsorption reshapes pathway selection, showing that protein layers can mask ligand–receptor interactions and suppress RMT targeting or promote opsonization-driven uptake, thereby modulating the balance between RMT, AMT, and nonspecific internalization routes [42].

These molecular-level simulations have involved simplified bilayers to sophisticated models that incorporate ligand–receptor binding, membrane curvature energetics, electrostatic cooperativity, and protein corona effects. They provide mechanistic insights and quantitative parameters that are increasingly integrated into higher-scale predictive frameworks.

#### Descriptor-based quantitative models

Descriptor-based models employ QSAR/QSPR approaches to relate NP physicochemical descriptors such as size, charge, surface chemistry, and protein corona composition with measurable biological endpoints, including cellular uptake and cell viability. Originally developed for small-molecule drug design, QSAR models quantify statistical links between molecular structures and bioactivities like enzyme inhibition (IC_50_), receptor affinity, cytotoxicity, and antimicrobial efficacy [43,44], while QSPR models predict physicochemical traits such as solubility, log P, polarity, melting point, and solvation free energy [45].

Within the framework of NP–BBB transport prediction, these 2 approaches converge into a unified continuum. Intrinsic NP features (e.g., core size, surface charge, and coating) align with QSPR domains, while their downstream effects on cellular interactions and transport dynamics fall under QSAR. This integration enables predictive modeling of NP behavior in complex biological environment, where protein corona formation dynamically modulates the NP’s “biological identity” [46]. Key early studies have established that quantitative profiles of adsorbed serum proteins, termed corona fingerprints, consistently outperform traditional physicochemical descriptors alone in predicting NP–cell association, with predictive accuracy increased by up to 50% across chemically diverse NP libraries [46,47].

Recent work has extended the classical QSPR framework into the concept of *nano-QSPR*, which integrates descriptors related to the NP core, surface coating, and protein corona to quantitatively model how their complex interactions determine the zeta potential and overall behavior of NPs in biological environments [48]. These findings underscore that the biological identity of an NP is not solely influenced by its synthetic parameters but is dynamically reshaped by adsorbed biomolecules, thereby bridging classical QSPR (material structure) and QSAR (biological outcome) into a seamless nano-QSPR continuum [47–50].

This descriptor-driven strategy offers practical advantages for developing brain-targeted nanomedicines. First, it provides interpretable, experimentally grounded models that outperform physics-based simulations in capturing the complex variability of biological environments. Second, it enables high-throughput virtual screening of NP libraries long before synthesis, reducing the experimental burden inherent to BBB transport studies. Third, by revealing specific corona proteins that either promote or suppress endothelial association, it opens rational routes to “corona engineering”. For instance, NPs can be precoated with proteins to help them cross the barrier. These advances turn the protein corona from an unpredictable problem into a useful tool for improving brain delivery. Table 8 summarizes representative QSAR/QSPR studies applying descriptor-based modeling to NP behavior.

**Table 8.** Representative descriptor-based (QSAR/QSPR) modeling studies

Study/Year	Modeling target	Input features	Algorithm(s)	Key insight
Walkey et al. (2014) [46]	NP–cell association (uptake)	NP size, surface charge, protein corona fingerprints	PLSR (with SFS-selected proteins)	Introduced corona-based QSAR paradigm: biological activity (cell uptake) predicted from corona fingerprints and physicochemical features.
Liu et al. (2015) [47]	NP–cell interaction intensity (MFI uptake)	Physicochemical descriptors and corona proteins	SVR (linear and nonlinear), linear regression (with SFFS)	Hybrid QSPR–QSAR model linking NP intrinsic properties (QSPR) and protein corona composition (QSAR) for quantitative uptake prediction.
Palchetti et al. (2016) [50]	Uptake classification (NP–cell binding categories)	Hard-corona protein fingerprints	PLSR-basedpredictive–validation modeling	Pure QSAR model using biological identity descriptors; protein family signatures act as activity determinants.
Bigdeli et al. (2016) [49]	Liposome–cell uptake and viability	Liposome size, surface charge, lipid type, protein corona composition, protein load	Linear and nonlinear QSAR models	Predominantly QSPR model with environment-adjusted parameters; integrates corona effects to refine physicochemical predictions.
Sengottiyan et al. (2023) [48]	Zeta potential prediction in biological media	Core, coating, and protein corona descriptors	GA–PLS nano-QSPR model	Defines the *nano-QSPR* framework bridging QSPR (material structure) and QSAR (biological outcome).

GA, genetic algorithm; GA–PLS, genetic algorithm–partial least squares; MFI, mean fluorescence intensity; PLSR, partial least-squares regression; QSAR/QSPR, quantitative structure–activity/property relationship; SFFS, sequential floating forward selection; SFS, sequential forward selection; SVR, support vector regression

#### Data-driven AI models

Data-driven artificial intelligence (AI) approaches, including ML and DL, have emerged as transformative methodologies for modeling NP–biological interactions, particularly in the complex domain of BBB transport. Recent reviews in nanomedicine and nanotoxicology highlight that AI/ML is well suited to capture nonlinear, multiscale relationships between NP design parameters, biological environments, and safety or efficacy outcomes, and to integrate heterogeneous experimental, simulation, and literature-derived data into predictive models [51,52]. AI models have also become a core strategy for predicting BBB permeability of small molecules and biologics, using physicochemical descriptors, graph-based encodings, and deep neural networks to infer transport across in vitro and in vivo BBB models [53–55]. These developments motivate the application of data-driven AI methods to NP transport, where the interplay between NP properties, protein corona formation, and cellular or barrier responses is even more multidimensional than in small-molecule systems. As summarized in Table 9, a total of 12 representative studies moved beyond basic outcome prediction toward a broader range of approaches, including improved ensemble models, richer descriptor design, and new directions such as causal analysis, hybrid simulation–ML frameworks, and image-based DL.

**Table 9.** Representative data-driven AI models

Study/Year	Modeling target	Input features	Algorithm(s)	Key insight
Cenk et al. (2014) [56]	NP uptake ratio over time	NP type, size, surface charge, concentration, incubation time	ANN	Early ANN model for NP interaction prediction.
Papa et al. (2016) [57]	Cellular association	Protein corona composition	PPR, MLR, PLS, SVM, NN, RF	Nonlinear ML (especially RF/SVM) far outperforms linear models; corona dominates uptake.
Findlay et al. (2018) [62]	Protein corona enrichment (binary)	Protein biophysical characteristics, NP features, and solvent conditions	Random Forest Classifier	RFC reveals protein biophysical properties weighted more than NP properties.
Duan et al. (2020) [63]	Protein corona composition (RPA)	Fluorescamine fluorescence change, NP properties	Random Forest	Novel fluorescence-based descriptor dramatically improves corona prediction across diverse NPs.
Ban et al. (2020) [58]	Functional corona composition → cellular recognition	NP properties and meta-analysis of corona proteomics	Random Forest	Multioutput ML model links corona functional classes (e.g., immune, complement, apolipoproteins) to cellular response.
Ouassil et al. (2022) [64]	Protein adsorption to CNTs (binary)	Protein sequence-derived properties	Random Forest Classifier	Corona formation predictable directly from amino acid sequence patterns without NP properties.
Fu et al. (2024) [59]	Multiprotein RPA in corona	Proteomics of 178 proteins and NP conditions	ERT, RF, XGBoost, LightGBM, NN	Tree ensembles achieve comprehensive RPA prediction for dozens of proteins simultaneously.
Bilgi et al. (2024) [60]	Cellular uptake of gold NPs	NP size, charge, dose, time, cell type	XGBoost, RF, LightGBM	ML highlighted size, surface charge, and exposure time as dominant factors.
Liao et al. (2024) [61]	Protein corona RPA	NP properties and plasma/experimental conditions	Random Forest	Resampling embedding resolves severe class imbalance, improving RPA prediction accuracy.
Yang et al. (2023) [67]	Single-cell NP uptake quantification	Fluorescence microscopy images	CNN (Cellpose)	Deep-learning segmentation enables high-throughput, single-cell uptake versus cell size.
Iaquinta et al. (2024) [66]	Membrane wrapping degree (uptake)	NP aspect ratio, adhesion, membrane tension	ANN surrogate of physics-based model	Hybrid mechanistic–ML framework revealed NP aspect ratio and initial adhesion are the dominant factors driving uptake.
Rafieioskouei et al. (2025) [65]	Causal drivers of corona diversity	Physicochemical and plasma composition features	Causal inference algorithm (Halpern–Pearl actual causality)	Causal model identified specific molecular drivers of corona formation beyond correlation.

ANN, artificial neural network; LightGBM, light gradient boosting machine; ML, machine learning; NN, neural network; NP, nanoparticle; PPR, projection pursuit regression; RF, random forest; RPA, relative protein abundance; SVM, support vector machine; XGBoost, extreme gradient boosting

One major type of research improves predictive performance by replacing linear models with adaptive, nonlinear algorithms capable of capturing complex NP–cell associations. For instance, neural-network models demonstrated the feasibility of learning NP–cell uptake dynamics directly from physicochemical descriptors and exposure conditions [56]. Early benchmarking showed that methods such as support vector machine (SVM) and random forest (RF) substantially outperform partial least squares (PLS), establishing nonlinear learning as the preferred strategy [57]. Building on this, tree-based ensemble methods have become widely adopted due to their robustness and interpretability [58–60]. Advanced ensembles such as XGBoost, LightGBM, and extremely randomized trees (ERT), often combined with SHAP (SHapley Additive exPlanations), have been used to predict cellular uptake and corona abundance while quantifying the influence of individual features, consistently identifying NP size, surface charge, and exposure conditions as dominant drivers [59,60]. Addressing challenges in biological datasets, resampling techniques integrated with RF classifiers have further improved prediction of corona composition under strong class imbalance [61].

These algorithmic improvements set the stage for incorporating richer biological descriptors. One major direction integrates high-dimensional proteomic or protein-derived features, including protein biophysical properties that strongly influence corona enrichment [62], detailed corona signatures linked to functional protein classes and cellular recognition [58], and relative abundance profiles of large protein panels for multiprotein corona prediction [59]. Other studies draw on additional biological signals, such as fluorescence change measurements from protein nanomaterial interaction assays [63], or amino acid sequence patterns that characterize adsorption behavior directly from protein sequences [64].

The most recent research employs sophisticated architectures to handle causality and unstructured data. Moving from correlation to causation, causal inference algorithms (e.g., Halpern–Pearl causality) have been applied to disentangle the true molecular drivers of corona formation from confounding variables [65]. Bridging data-driven and mechanistic worlds, hybrid frameworks using artificial neural networks (ANNs) as surrogates for mechanical simulations have been proposed, allowing for rapid prediction of uptake wrapping degrees constrained by physical laws [66]. DL represents a more recent advancement, enabling direct feature extraction from complex or multimodal data without predefined descriptors. Convolutional neural networks (CNNs) have been used to analyze fluorescence microscopy images and identify NP uptake patterns at the single-cell level [67]. Although DL applications remain limited within BBB-specific systems, their capacity for automated representation learning indicates strong potential for integrating structural, proteomic, and imaging data into unified predictive frameworks.

Overall, ML/DL evolve QSAR/QSPR into adaptive systems for NP–bio modeling, with these 12 studies predicting corona, uptake, and transport. While datasets emphasize general assays, these methods pave the way for BBB-specific multimodal integration, accelerating brain-targeted NP design.

#### System-level kinetic models

System-level kinetic modeling of NPs, including PK/PD and PBPK simulations, provides a framework to predict NP transport and distribution across biological compartments. These models extend beyond atomistic or cellular-scale observations by using systems of ordinary differential equations (ODEs) or compartmental PBPK structures to simulate time-dependent concentration and distribution profiles, particularly between plasma and brain, which directly reflect BBB permeability [68,69].

In contrast to molecular- or cell-level simulations, PK/PD and PBPK approaches operate at the whole-body scale, linking physicochemical determinants such as size, charge, and surface chemistry to systemic pharmacokinetics and therapeutic outcomes. They integrate in vitro permeability assays, in vivo biodistribution data, and mechanistic kinetic constants to reproduce real physiological transport.

For example, receptor-mediated endocytosis [70] and AMT [71] have been mathematically represented within PBPK frameworks, allowing estimation of brain exposure and tissue-specific accumulation. Similarly, integrated hybrid models combine experimental PK data with simulated parameters like permeability and clearance to validate transport mechanisms for nanocarriers [72–74].

These models provide the most direct quantitative link between experimental data and systemic pharmacokinetics. With proper experimental calibration, they can predict how NPs distribute across organs and identify design factors such as ligand type, coating, and receptor affinity that improve BBB permeability. PBPK and PK/PD simulations therefore serve as a mechanistic bridge between nanoscale interactions and whole-body drug distribution, helping to guide the design of BBB-targeted nanocarriers, as summarized in Table 10.

**Table 10.** Representative PK/PD and PBPK modeling studies

Study/Year	Surface ligand or modification	Targeted BBB mechanism	Modeling context
Riviere et al. (2018) [74]	Apolipoprotein E (ApoE) mimetic peptide	Interaction with LDL receptor-related proteins (LRP1/LRP2)	PBPK model describing biodistribution of gold NPs; highlighted coating- and corona-dependent partitioning across organs, though not explicitly BBB-specific.
Deng et al. (2019) [70]	Transferrin (Tf)	Receptor-mediated transcytosis via transferrin receptor (TfR)	PBPK framework incorporating receptor-mediated endocytosis kinetics to simulate NP uptake, intracellular accumulation, and brain exposure.
Hu et al. (2019) [72]	Lactoferrin (Lf)	Receptor-mediated uptake through lactoferrin receptors on brain endothelium	PK/PD model integrating BBB permeability ($K_{p,\mathrm{uu}}$) and therapeutic outcome to evaluate Lf-nanocarrier delivery efficiency.
Kadakia et al. (2019) [73]	Insulin/insulin-like peptide	Insulin receptor-mediated transcytosis	Mathematical transport model for intranasal nanoemulsion; validated BBB crossing efficiency against experimental PK data.
Abd-Algaleel et al. (2021) [71]	Cationic coating (chitosan, TAT peptide)	Adsorptive-mediated transcytosis via electrostatic interaction	Integrated in silico–in vitro–in vivo PBPK model quantifying permeability and nose-to-brain transport kinetics.

BBB, blood–brain barrier; LDL, low-density lipoprotein; NP, nanoparticle; PBPK, physiologically based pharmacokinetic; PK/PD, pharmacokinetic/pharmacodynamic

#### Nanoinformatics frameworks

Nanoinformatics seeks to establish an integrated digital ecosystem for managing, structuring, and utilizing diverse NP data. Unlike traditional methods that focus on individual simulations or experiments, nanoinformatics frameworks operate at a higher level of abstraction. They curate, standardize, and interlink different types of information, including physicochemical properties, protein corona profiles, mechanistic simulation outputs, and in vitro uptake measurements. This comprehensive approach enables downstream modeling, model validation, and data-driven design of NPs. Core components typically include harmonized ontologies, FAIR (Findable, Accessible, Interoperable, and Reusable)-compliant workflows, high-quality metadata capture, and platforms that enable machine-readable representations of nano–bio interactions. A primary function of nanoinformatics is data integration. Fragmented datasets, including physicochemical characterization, corona proteomics, cellular uptake assays, PK/PD measurements, and simulation-derived descriptors, are transformed into interoperable formats through standardized pipelines. Large-scale resources such as Walkey et al. [46] and Ban et al. [58] demonstrate how data mining and knowledge discovery can transform aggregated experimental datasets into predictive models and reveal transferable nano–bio interaction patterns.

Among data-driven approaches, 2 studies explicitly introduced the idea of nanoinformatics features and workflows. One example is a KNIME-based QNAR pipeline [75], which integrates preprocessing, feature selection, model validation, and applicability domain assessment into a single decision-support system that enables virtual screening of NPs using protein corona fingerprints. The final models and datasets were released on the Enalos Cloud Platform as an open web tool, showing how curated workflows can turn experimental information into accessible and reusable digital resources. A second example is a combined in vitro and in silico workflow [76]. Experimental protein binding assays, UnitedAtom corona predictions, and CoronaKMC simulations were organized and annotated with nanosafety ontology terms, and the full set of data and metadata was deposited in the NanoCommons Knowledge Base under FAIR principles. This work highlights the importance of complete metadata, interoperability across tools, and iterative model refinement guided by experimental benchmarking, which are central goals in modern nanoinformatics.

These 2 works show how nanoinformatics increases transparency, improves cross-study comparability, and enables scalable data reuse by encouraging clear documentation, standardized metadata, and open access to datasets and computational workflows. By integrating corona fingerprints, physicochemical descriptors, and mechanistic simulation outputs into unified and interoperable infrastructures, nanoinformatics establishes a solid foundation for multimodal predictive modeling across the early stages of NP transport, particularly corona formation and cellular association. As the field continues to develop, such infrastructures will be essential for building larger and more diverse NP libraries, incorporating emerging BBB-on-chip measurements, supporting AI-driven feature discovery, and advancing next-generation computational models for BBB transport.

## Cross-Scale Comparative Analysis of In Silico Approaches

This section provides a cross-scale comparative analysis of existing in silico approaches, examining how NPs and BBB components are represented, which datasets support these modeling efforts, and how different computational methods operate across molecular, mesoscopic, cellular, and system-level scales.

### Feature representations

Representing NPs and BBB components for modeling remains challenging because both are complex, multicomponent systems that span molecular, cellular, and physiological scales. An NP consists of a core material, surface coating, targeting ligands, and a dynamic protein corona, while the BBB is a multicellular interface that regulates selective transport through multiple mechanisms. Mechanistic understanding therefore guides the selection of descriptors that capture relevant structure–property relationships. In this review, 5 complementary descriptor tracks were identified, which together encompass the vast majority of feature representations used across computational NP–BBB studies. These tracks can be engineered and combined into multimodal feature spaces:

1. Physicochemical descriptors: NP intrinsic properties such as core material, size, shape (e.g., aspect ratio), ζ potential, coating chemistry, hydrophobicity, ligand density, polydispersity index, concentration, and exposure time [46,47,49,56–61].
2. Computational and topological encodings: Machine-readable representations of NP structure and bio-interaction context, including quasi-SMILES strings that encode core–coating–corona interactions [48], and high-dimensional morphological and uptake features extracted via DL from fluorescence microscopy images [67].
3. Biological corona fingerprints: Descriptors representing the biological identity of NPs, including protein fingerprints [46,62,63], relative abundance of key protein markers [47,49,57], sequence or structural features of corona proteins [64], and corona dynamics categories [50].
4. Mechanistic and transport-related parameters: Descriptors quantifying the physical and physiological mechanisms that govern NP interactions and transport, including interaction or adhesion energy, membrane mechanics (bending rigidity, tension, and wrapping fraction), diffusion and permeability coefficients, receptor binding rates, and endocytosis or transcytosis kinetics [66,70–74]. Experimental and environmental factors such as plasma concentration, incubation time, temperature, centrifugation speed, and exposure duration [56,59–61,63].
5. System kinetic variables: Descriptors derived from PK/PD or PBPK models, including systemic compartmental parameters such as clearance (CL), distribution volume ($V_{t}$), and vascular extraction rate that capture global transport behavior [70,74]; partitioning and permeability variables such as brain–plasma partition ratio ($K_{p,\mathrm{uu}}$), permeability constants ($P_{\mathrm{BBB}}$), and nasal bioavailability ($F_{N}$) describing multipathway BBB transport [72,73]; and dynamic exposure parameters such as drug release rate ($k_{\mathrm{rel}}$), drug targeting efficiency, and direct transport percentage, which represent targeting efficiency [71,72].

By integrating standardized physicochemical metrics with emerging computational encodings, current modeling frameworks can now capture both explicit experimental properties and implicit structural patterns, enabling more robust predictions of NP transport across the BBB.

### Key datasets and reuse

To clarify the evidential base for NP–BBB modeling, we also summarize representative datasets and their reuse. Analyzing the spread of widely used resources across studies helps identify opportunities for harmonization and benchmarking, while also exposing biases in data availability and selection. Among the reviewed studies, 2 foundational datasets have strongly influenced predictive modeling of NP–cell interactions: the primary experimental dataset by Walkey et al. [46] and the meta-dataset compiled by Ban et al. [58]. Walkey et al. introduced protein–corona fingerprinting through a systematically characterized primary dataset of 105 Au/Ag NPs, linking quantified corona profiles to matched cellular–association measurements and demonstrating that corona composition predicts NP–cell interactions. Ban et al. expanded this concept by compiling a literature-derived meta-dataset (652 samples, 178 proteins) harmonizing heterogeneous corona measurements across studies. These datasets have been widely reused in subsequent QSAR and ML studies [47,57,59,61,75], which applied regression, ensemble learning, and feature-importance analysis to refine corona prediction and identify key mechanistic determinants. Complementary datasets further expanded NP representations through diverse data modalities relevant to BBB transport, including secondary reanalysis of existing proteomics datasets [48,62,64], primary experimental datasets generated from new corona or uptake measurements [49,50,56], literature-derived or meta-analytic datasets [60,63], high-throughput imaging datasets enabling single-cell uptake quantification [67], simulation-derived mechanistic datasets [66], and hybrid datasets integrating in vitro and in silico corona predictions [76]. Where BBB-specific labels are absent, these resources support proxy tasks (e.g., corona composition and uptake) and descriptor transfer into permeability or transcytosis models. Representative datasets and their reuse relationships are summarized in Table 11.

**Table 11.** Representative datasets and their reuse relationships between studies

Study	Dataset role/Origin	Modeling focus
Walkey et al. (2014) [46]	Primary experimental (105 Au NPs; proteomics)	Cellular uptake prediction: Developed predictive model linking Au/Ag NP corona fingerprints with cellular association.
Liu et al. (2015) [47]	Secondary reanalysis (subset: 84 NPs)	Cellular uptake prediction: Applied nonlinear SVR with feature selection; identified minimal feature set for improved accuracy (*R*^2^ = 0.895)
Papa et al. (2016) [57]	Secondary reanalysis (subset: 84 NPs)	Cellular uptake prediction: Comp ML methods (PPR, RF, SVM, etc.) with rigorous external validation
Afantitis et al. (2018) [75]	Secondary reanalysis (full set: 105 NPs)	Cellular uptake prediction: Developed a k-Nearest Neighbor (kNN) model and deployed it as a web service (Enalos Cloud Platform) for public virtual screening and decision support.
Ban et al. (2020) [58]	Primary meta-dataset (652 samples; 178 proteins)	Protein corona prediction: Constructed large-scale dataset via literature mining; used Random Forest (RF) to predict functional corona composition and cellular recognition.
Fu et al. (2024) [59]	Secondary reanalysis (full ban set)	Protein corona prediction: Proposed 2-stage prediction (classification + regression); added SHAP interpretability.
Liao et al. (2024) [61]	Secondary reanalysis (add new experimental validation)	Protein corona prediction: Applied resampling techniques (e.g., SMOTE) to resolve data imbalance; validated model with new label-free quantification of 4 NPs.
Findlay et al. (2018) [62]	Secondary reanalysis [107]	Protein corona prediction: Used Random Forest models to classify Ag NP corona membership (PC vs. non-PC) based on protein abundance, NP size, and surface chemistry.
Ouassil et al. (2022) [64]	Secondary reanalysis [108]	Protein corona prediction: Trained a sequence-based Random Forest Classifier to predict protein adsorption to carbon nanotubes in plasma and CSF, using amino acid and surface-exposure descriptors.
Sengottiyan et al. (2023) [48]	Secondary reanalysis [109]	Physicochemical modeling: Developed GA–PLS models for Zeta potential (ζ) prediction; emphasized the combined role of core, coating, and corona descriptors.
Bigdeli et al. (2016) [49]	Primary experimental dataset	Cellular uptake prediction: Combined liposome corona fingerprints and physicochemical descriptors to model uptake and cytotoxicity in PC3/HeLa cells.
Palchetti et al. (2016) [50]	Primary experimental dataset	Cellular uptake prediction: Identified a specific set of 8 corona proteins (e.g., Vitronectin, ApoA1) as the main drivers for NP–cell association.
Duan et al. (2020) [63]	Secondary (literature-derived)	Protein corona prediction: Developed novel protein descriptors and Random Forest models to predict relative protein adsorption on diverse nanomaterials.
Bilgi et al. (2024) [60]	Secondary (systematic meta-analysis)	Cellular uptake prediction: Large-scale meta-analysis of Au NP cellular uptake; XGBoost model identified exposure time, concentration, and zeta potential as key drivers.
Cenk et al. (2014) [56]	Primary experimental dataset	Cellular uptake prediction: ANN-based modeling of uptake rates over 48 h; simulated time-dependent interactions to reduce experimental cost.
Yang et al. (2023) [67]	Primary experimental imaging dataset	Deep learning segmentation: Utilized Cellpose algorithm to quantify single-cell NP uptake from microscopy images, creating a large-scale uptake dataset.
Iaquinta et al. (2024) [66]	Primary simulated dataset	Mechanistic uptake modeling: Generated 4,096 synthetic samples to model the membrane wrapping process of elliptical NPs using ANN sensitivity analysis.
Rafieioskouei et al. (2025) [65]	Primary experimental dataset	Physicochemical modeling: Used GA–PLS regression with core, coating, and corona descriptors to predict NP zeta potential and map applicability domains.
Hasenkopf et al. (2022) [76]	Hybrid dataset (primary experimental and simulated output)	Protein corona modeling: Validated in silico corona prediction tools (UnitedAtom) against in vitro data; deposited FAIR-compliant datasets to NanoCommons.

ANN, artificial neural network; CSF, cerebrospinal fluid; FAIR, Findable, Accessible, Interoperable, and Reusable; GA–PLS, genetic algorithm–partial least squares; ML, machine learning; NP, nanoparticle; PPR, projection pursuit regression; RF, random forest; SHAP, SHapley Additive exPlanations; SVM, support vector machine; SVR, support vector regression; XGBoost, extreme gradient boosting

When it comes to data availability, which is crucial for ensuring that research can be reproduced and independently verified, there is considerable variation across the reviewed studies. Most QSAR/QSPR, ML, and nanoinformatics studies achieve full or near-full openness (score 2). Authors routinely share complete datasets, preprocessing scripts, trained models, and source code on public repositories such as Zenodo or GitHub, and several even provide freely accessible web tools (e.g., Enalos Cloud) that include both original and extended results [75]. Stand-out examples include the works of Afantitis et al. and Hasenkopf et al. [75,76], which deliver ready-to-use predictive models alongside cloud-hosted platforms, comprehensive Modeling and Data Analysis (MODA) documentation, and fully archived workflows. The only exceptions in this category are Refs. [49,56], where proprietary experimental data prevented complete release despite otherwise transparent methodology. In contrast, all 5 PK/PD modeling studies exhibit moderate to low openness (score 1) [63,70,72–74]. Although they provide detailed mathematical equations, compartment diagrams, and literature-derived parameters, none share actual model implementation code, input files, or raw simulation datasets. Reproducibility is therefore limited to reimplementation from scratch.

Simulation-based studies show the broadest range of practices but generally trend toward lower practical accessibility. Multiscale and hybrid/enhanced-sampling simulations achieve the highest openness in this group (score 2). Excellent examples are provided by Power et al. and Schneemilch and Quirke [77,78] and Subbotina et al. [79], who deposit complete archives of GROMACS input files, PLUMED scripts, custom force-field parameters, and truncated trajectories on Zenodo or publisher repositories. The UANanoDock platform [79] further stands out by offering a permanent web interface with all underlying Python code openly available on GitHub. Pure AAMD, CGMD, and DPD studies, however, mostly remain at score 1: they describe methods thoroughly but almost never release downloadable input files or full trajectories, primarily because of their large size and complexity. A complete table summarizing data-availability scores and supporting evidence for all reviewed studies is provided in the Supplementary Materials.

### Modeling resolution and computational scales

Modeling approaches for NP transport across the BBB operate over markedly different spatial and temporal scales, each capturing distinct stages of the overall process.

#### Molecular and mesoscopic simulation scales

AAMD and CGMD operate at the highest resolution (approximately angstroms to nanometers in space and nanoseconds to microseconds in time) and dominate the investigation of early physicochemical events, including protein corona formation (Stage 1) and initial NP–membrane recognition (Stage 2).

AAMD represents every atom explicitly and employs classical force fields (e.g., AMBER and OPLS) to follow Newtonian dynamics [80]. In the reviewed literature, AAMD has been applied to (a) lipid-based and PEGylated liposomes [81], (b) charged or functionalized metal NPs under varying ionic conditions [82], (c) conformational flexibility of BBB-shuttling peptides [83], and (d) drug encapsulation within polymeric nanocarriers [84]. These studies deliver quantitative adsorption free energies, hydrogen-bonding networks, and local bilayer deformation. Importantly, AAMD outputs routinely serve as reference data for parameterizing higher-scale models in subsequent multiscale simulation works [77,78,85–87].

CGMD operates at mesoscale resolution (a few nanometers to hundreds of nanometers, microseconds to milliseconds) and most frequently uses the MARTINI force field [88]. By grouping atoms into beads, it sacrifices atomic detail but enables simulations of much larger systems and longer timescales than AAMD. Across NP–BBB studies, CGMD has been widely applied to model protein corona formation (Stage 1), NP–membrane binding (Stage 2), and receptor-mediated uptake (initial Stage 3). Representative CGMD studies have revealed how surface chemistry, charge, and ligand flexibility regulate cooperative protein adsorption [39,41,89,90]. For example, preadsorbed serum albumin or flexible ligands such as PDADMAC can modulate corona formation and stability through altered electrostatic and hydrophobic interactions [39,89]. Additionally, NP elasticity plays a key role in receptor-mediated membrane wrapping [37]: softer NPs attach more rapidly in the early stage because deformation increases the contact area with the membrane, yet they are engulfed more slowly in the late stage due to the higher energy cost of deformation. This nonmonotonic behavior reproduces the experimentally observed dependence of cellular uptake efficiency on NP stiffness.

Mesoscopic approaches such as DPD and multiscale or hybrid simulations extend this resolution by several orders of magnitude, reaching hundreds of nanometers and microsecond to millisecond regimes. They capture cooperative wrapping, receptor-mediated endocytosis, and collective membrane deformation, thereby bridging molecular detail with emergent mesoscale dynamics. Across the reviewed studies, DPD has been applied to multiple mechanistic stages of NP transport across the BBB. At the protein corona formation stage, NP hydrophobicity was modeled to tune corona microstructure [91], and corona formation was further linked to subsequent cellular uptake [42]. At the NP–membrane interaction stage, several studies explored how NP charge, shape, and elasticity regulate adhesion and wrapping [34,36,92,93]. Finally, at the cross-BBB transport stage, receptor-mediated endocytosis and cooperative internalization were simulated to derive scaling relations between NP physicochemical parameters and uptake kinetics [35,38,94]. Together, these studies reveal a consistent mesoscale picture of NP adsorption, wrapping, and internalization. They are methodologically transparent and qualitatively validated but use simplified, non-BBB membranes.

By coupling atomistic potentials with continuum or coarse-grained models, multiscale or hybrid simulations enable partial connection between early molecular interactions and the onset of cellular uptake. These frameworks combine AAMD precision with CGMD efficiency, linking molecular features (e.g., binding energy, curvature response, and elasticity) to experimental readouts (e.g., corona composition and uptake efficiency). Across the reviewed studies, multiscale modeling has been applied mainly in 2 directions. The first focuses on protein corona formation, where CGMD captures cooperative adsorption and restructuring while AAMD refines atomistic interactions and energetics [77,79,85–87]. The second addresses NP–membrane adhesion and uptake, linking molecular adhesion strength to larger-scale wrapping behavior [78]. Representative examples illustrate this complementarity: CGMD and AAMD were combined to simulate sequential protein adsorption on gold NPs, showing how cooperative interactions and local relaxation drive corona restructuring [87]; peptide adsorption on silver NPs was modeled using CGMD followed by atomistic refinement, confirming curvature-dependent peptide orientation consistent with experimental data [85]; atomistic, coarse-grained, and continuum thin-film models were integrated to predict NP wrapping efficiency with strong quantitative validation and transparency [78]; and this concept was extended into a web-based multiscale docking tool (UANanoDock) with fully open data and high reproducibility [79].

Hybrid and enhanced-sampling approaches extend MD by adding continuum, electrostatic, or external-field models, or by accelerating rare molecular events through biasing techniques. At the protein corona formation stage, hybrid electrostatic models combining explicit-solvent MD with continuum frameworks showed that ligand patterning and charge regulation control interaction strength between proteins and functionalized NP surfaces [95,96]. At the NP–membrane interaction stage, metadynamics and umbrella sampling reveal free-energy landscapes of adsorption and penetration: silica–membrane adhesion energies were computed using well-tempered metadynamics [97], while other studies quantified how NP size, charge, and surface chemistry affect energy barriers and uptake probability [40,98]. At the cross-BBB transport stage, field-coupled simulations (e.g., magnetic guidance) explored how external forces influence NP motion through endothelial membranes; steered MD was used to calculate force profiles and diffusion coefficients, identifying magnetic field strengths consistent with experimental limits and BBB permeability data [99–101]. Together, these enhanced-sampling and hybrid continuum-electrostatic methods provide accurate free-energy barriers for membrane penetration and translocation—information that is difficult to obtain from standard simulations.

Overall, these physics-based methods provide increasing system size and temporal coverage, while gradually sacrificing atomistic detail. However, most current simulations still rely on simplified membrane models and do not include key BBB features such as tight junctions, glycocalyx, efflux pumps, or realistic flow. As a result, they offer strong mechanistic insight but remain limited in biological realism.

This limitation is reflected in how the BBB is typically represented, often as a simplified membrane rather than a structured neurovascular interface. Accordingly, current MD-based studies capture important nanoscale interaction mechanisms but still exhibit limited physiological realism at the endothelial barrier level. In practice, this simplification appears as the representation of the BBB as a lipid membrane or effective endothelial interface rather than a fully structured neurovascular barrier. Explicit tight junction architectures are generally not modeled; instead, the barrier is approximated as a continuous membrane lacking claudin/occludin complexes and paracellular sealing organization [100,101]. Similarly, the endothelial glycocalyx is rarely included as a spatially resolved carbohydrate-rich surface layer, with interfacial biology typically represented indirectly through adsorbed proteins, hydration shells, or synthetic surface coatings [85,86].

Receptor-mediated processes are also commonly simplified. Many studies implement ligand–receptor interactions using uniform receptor densities or idealized binding rules, without accounting for heterogeneous receptor distributions, clustering, membrane polarity, or intracellular trafficking constraints that characterize BBB endothelium [34,36]. While some simulations explore variations in lipid composition, membranes are usually modeled with homogeneous or low-complexity phospholipid mixtures, and BBB-specific lipid asymmetry and transporter-rich microdomains remain largely absent [40].

At the dynamic level, simulations typically resolve local membrane responses such as adsorption, lipid rearrangement, transient pore formation, and NP wrapping, but they rarely capture larger-scale endothelial remodeling processes, including cytoskeleton coupling, vesicular maturation, polarized transcytosis, or multicellular barrier organization [37,97].

#### Data-driven and system-level modeling scales

In contrast, descriptor-based and data-driven models abstract these physical interactions into statistical or functional relationships. At these levels, modeling spans cellular dimensions (1 to 50 m) and extends to systemic, organ, or whole-body scales (centimeters), capturing uptake behavior and organism-level pharmacokinetics.

Cellular-scale QSAR/ML models generalize these mechanistic interactions to predict population-level uptake and biological responses, leveraging corona fingerprints, physicochemical descriptors, and high-throughput imaging. These models target the integrated phenotype of entire cell populations rather than single-particle trajectories. Across multiple studies, corona-derived descriptors were shown to systematically outperform intrinsic physicochemical properties for predicting NP–cell association [46,47,49,50]. Many ML frameworks further revealed that immune-, complement-, apolipoprotein-, and adhesion-related protein families act as key molecular mediators bridging corona formation (Stage 1) and cellular uptake (Stage 2) [57–59,61,62]. High-throughput imaging integrated with DL expands this cellular-level perspective: a Cellpose-based workflow enables quantification of single-cell NP uptake across thousands of cells, exposing heterogeneity not resolvable by MD or DPD simulations [67].

System-scale PK/PD and PBPK models combine cellular-level processes into whole-body transport and correspond to Stage 3 (cross-BBB transport). They use ODEs to describe how NP concentrations change across organs over hours or days. By incorporating design-related parameters such as permeability, ligand density, charge, and release rate, they link NP properties to in vivo biodistribution and brain exposure [72–74].

Several extensions improve the biological realism of these system-scale predictions. One important direction integrates endocytosis mechanisms directly into PBPK equations, allowing receptor binding and intracellular trafficking to influence organ-level distribution [70]. Other models include alternative delivery routes, such as nasal administration, to estimate nasal bioavailability and nose-to-brain transport [71]. Flow-based simulations describe vascular extraction and tissue deposition driven by blood movement, capturing hemodynamic factors that molecular-scale simulations cannot resolve [74]. Additional parameters, including diffusion, partitioning, and endothelial permeability, further connect nanoscale physicochemical properties to predictions of BBB permeability and CNS exposure [73]. Together, these cellular- and system-scale approaches extend in silico BBB modeling beyond atomistic and mesoscale limits. They demonstrate how molecular interactions and cell-level uptake ultimately determine whole-body NP distribution and brain delivery.

In this context, data-driven and system-level models focus on broader biological patterns. QSAR and ML summarize complex interactions into predictors of uptake, while PK, PD, and PBPK models connect cell-level processes to whole-body distribution. These approaches show a trade-off between physical realism and physiological scope, as they capture large-scale biology but still rely heavily on the quality of molecular and mesoscale data. This trade-off highlights the need for integration across modeling levels.

Across the reviewed QSAR and ML studies, a consistent pattern emerged regarding the biological scope of the modeled endpoints. Most data-driven models were developed to predict upstream NP–biological interactions such as protein corona composition or general cellular uptake. Training datasets were typically derived from plasma environments or non-BBB cell systems, including epithelial and cancer cell lines. While some studies incorporated endothelial interaction proxies or transport-related descriptors, none were explicitly trained or validated using BBB-relevant systems such as brain endothelial models, BBB-on-chip platforms, or in vivo brain distribution data. Consequently, these approaches primarily characterize NP bio-identity and interaction potential at the cellular level, rather than directly modeling BBB permeability or brain accumulation.

This points toward the need for integrated strategies in which atomistic parameters, mesoscale trends, and statistical models are combined with physiologically grounded system-level frameworks to improve predictions of BBB permeability and brain delivery.

#### Fragmentation across modeling scales

Consistent with the integration challenges discussed above, the modeling of NP transport across the BBB remains highly segmented. While experimental data can be obtained from in vitro and in vivo studies, the reviewed literature shows that current in silico models rely on several distinct types of experimentally derived datasets that span different biological scales.

At the molecular level, data are typically generated from protein–NP interaction experiments. In these studies, NPs are incubated in biological fluids and the adsorbed proteins are quantified using proteomics, producing relative abundance profiles that define the NP’s biological identity (“protein corona”) [46,49,50,57]. These corona fingerprints, often combined with physicochemical descriptors, are widely used to train QSAR or ML models that predict nano–bio interactions, corona composition, or biological responses [58,59,61,63,64].

At the cellular and tissue levels, NP–cell interaction datasets are typically obtained from in vitro exposure experiments quantifying cellular association, uptake, or bioactivity. Several studies integrate measured corona fingerprints and physicochemical properties with experimentally quantified cellular responses to develop predictive models of NP internalization or interaction behavior [47,60,67,75]. Such datasets establish mechanistic links between molecular-scale adsorption processes and cellular outcomes.

At the systemic level, pharmacokinetic and biodistribution data are derived from in vivo experiments, perfused tissue studies, or literature-based parameterization, measuring NP concentrations in blood, organs, or tissues over time. These measurements enable compartmental or PBPK models describing absorption, distribution, and elimination processes, often incorporating experimentally derived uptake or endocytosis parameters [70,72–74]. Such models provide a framework for linking NP properties to whole-body transport dynamics and therapeutic performance.

Despite the availability of these datasets, in silico simulations and data-driven models are often developed separately at each scale, using different assumptions, resolutions, and modeling goals. As a result, their outputs are difficult to combine into a single continuous transport sequence that spans molecular recognition, cellular interaction, trans-BBB transport, and systemic distribution. Even studies that align computational predictions with experimental workflows usually operate within limited scopes rather than offering a fully integrated multiscale framework [71,76].

This separation across data sources reinforces the lack of a unified in silico framework capable of capturing the full BBB transport process of NPs. Instead, existing studies focus on isolated mechanistic stages, leaving gaps between molecular, cellular, and organism-level models.

## Evaluation and Limitations

This section examines how current in silico models perform in practice by reviewing validation approaches, preprocessing strategies, and performance reporting across studies. It also highlights key limitations in reproducibility, biological realism, and data consistency that may affect predictive robustness and clinical relevance.

### Model performance and validation strategies

To assess the reliability of current data-driven models, we reviewed how the included studies handled data preprocessing, designed validation protocols, used external validation or experimental tests, and reported performance and interpretability metrics. These 4 components affect whether model predictions are robust, reproducible, and biologically meaningful.

In preprocessing and data curation, many studies showed that model performance depends on how input data are cleaned and standardized. For example, resampling methods such as SMOTE and random oversampling were essential for correcting imbalanced proteomic datasets, reducing root mean square error (RMSE) by nearly 10% in corona-regression tasks [61]. Standardization of heterogeneous experimental measurements, including harmonizing TEM and DLS size values and normalizing protein abundances, enabled the integration of large multisource datasets containing 652 samples [58]. Additional filtering steps, such as outlier removal and correlation-based feature reduction, were used to stabilize learning on small datasets and reduce noise in high-dimensional proteomic data [59]. Studies also used feature-selection strategies to improve stability and reduce overfitting. Some QSAR models applied manual or stepwise regression selection [46,50], while other works adopted algorithmic approaches such as sequential forward floating selection (SFFS) [47] and genetic algorithm–partial least squares (GA–PLS) for multidomain descriptor optimization [48].

During validation design, the reviewed studies showed clear differences in how rigorously data were split and tested. Stratified sampling was often used to preserve the distribution of rare high-affinity binders and to avoid biased training sets [59]. Several studies applied external hold-out validation. For example, one study kept 26 NPs completely unseen during training [75]. This provided a more realistic estimate of generalizability than leave-one-out cross-validation (LOOCV), because LOOCV can give overly optimistic results on small datasets [46]. Most models combined train/test splits with k-fold cross-validation or bootstrap resampling to reduce overfitting and improve model stability [57,61]. Leakage control was also important, especially when studies used shared proteomic or NP features, because leakage can artificially inflate performance.

After model training, several studies used external or experimental validation to test generalization. For example, one study trained a model on gold NPs and tested it on silver NPs to examine material-level transferability [47]. Some studies compared predicted protein-binding profiles with wet-lab measurements such as mass spectrometry and fluorescence assays to verify molecular-level interactions [63,64,76]. For image-based uptake prediction, deep-learning segmentation methods were benchmarked against manual annotations to support reliable single-cell statistics [67]. Mechanistic–ML surrogate models also compared uncertainty and sensitivity with physics-based simulations and found similar trends [66]. In simulation studies, validation often compared computed free-energy barriers or uptake rates with experimental fluorescence or TEM data, although such direct comparisons remain limited [78,79]. These external and experimental tests support biological relevance and reproducibility.

For model evaluation, most studies used standard regression and classification metrics to assess predictive performance. Tree-based ensemble models such as RF, gradient boosting, XGBoost, and LightGBM typically reached $R^{2}$ values above 0.75 for predicting corona composition and cellular uptake [58–60]. Regression tasks were mainly evaluated using $R^{2}$ and RMSE, while classification tasks relied on accuracy, area under the receiver operating characteristic curve (AUC), or F1 score. To understand the drivers of model predictions, a growing emphasis on explainable AI is evident in the reviewed studies, which increasingly employ embedded and post-hoc interpretability techniques to move beyond black-box predictions and extract biologically meaningful insights. Embedded approaches such as RF importance and mutual-information ranking identified influential descriptors including NP size, ζ potential, coating chemistry, and key corona proteins [59,60]. Post-hoc tools such as SHAP values, permutation importance, and partial dependence plots provided similar insights and helped visualize how these variables shaped the predicted outcomes [58,59]. More recent work has further advanced beyond correlation-based rankings by applying causal-inference techniques to identify molecular drivers of corona formation [65].

Across PK/PD and PBPK studies, validation followed a broadly consistent pattern. Models were typically calibrated on published rodent biodistribution or concentration–time data, with parameters such as permeability, endocytosis rates, and partition coefficients fitted to reproduce observed organ profiles [70,72,73]. Model performance was assessed mainly through visual agreement with experimental concentration curves, goodness-of-fit PK metrics (e.g., AUC and $t_{1/2}$), or sensitivity analyses identifying influential transport parameters [73,74]. Some studies also compared in silico predictions with experimental encapsulation efficiency or brain-targeting metrics after intranasal delivery [71]. Independent external datasets were rare. Many models relied on literature-derived parameters and did not report uncertainty. This limits validated forward prediction.

### Reproducibility, validation, and transparency

Across data-driven models and simulations, descriptor-level heterogeneity remains a key limitation for reproducibility and cross-study benchmarking. Core NP descriptors such as size, surface charge, and protein corona composition are often measured under differing experimental conditions (e.g., TEM vs. hydrodynamic size and plasma-dependent corona formation), resulting in context-dependent biological identities [46,47,49]. In addition, uptake and transport parameters used in modeling frameworks are frequently inferred from literature rather than measured under standardized conditions [70,72]. FAIR-oriented workflows have further highlighted inconsistencies and incomplete metadata in NP characterization datasets [76]. Together, these factors limit direct comparison across studies and underscore the need for standardized reporting.

In addition to data-driven models, molecular simulations also show substantial heterogeneity in how NP properties and BBB environments are defined. Studies use different membrane compositions, surface charge representations, and interaction parameters, often based on simplified or system-specific assumptions rather than standardized definitions. For example, many simulations rely on idealized lipid bilayers with limited compositional complexity [40,97], while key descriptors such as surface charge are interpreted differently depending on ligand density, electrolyte environment, or model formulation [95]. This variability limits cross-study comparability and supports the need for more harmonized modeling assumptions alongside standardized experimental descriptors.

Most simulation studies provide sufficient methodological details. They typically report force fields, system compositions, and simulation parameters [37,40,41,81,97]. However, many do not share input files, trajectories, or executable models in public repositories [39,83,84,89,92,99]. Only a smaller group of studies supports deeper reproducibility by sharing simulation inputs or multiscale tools [77–79].

In terms of validation, qualitative validation was the most common approach. Many studies checked whether their results matched known experimental trends or previous computational findings [81,83,84,89,92,99]. A smaller group of studies applied more quantitative validation methods. These included comparing predicted uptake behavior with experimental data [37], benchmarking free-energy results against literature data [97], and comparing internalization rates with known biological observations [40,98]. Some studies also validated their results against higher-resolution simulations or theoretical models [78,86].

A limited number of studies demonstrate stronger validation practices by combining predictive modeling with independent experimental confirmation. For example, imaging-based DL models have been validated against experimentally measured uptake behavior [67], while protein corona prediction frameworks have linked computational outputs with biological recognition outcomes [58]. Other studies explicitly integrate computational prediction with experimental analysis to test model reliability [76]. Multiscale modeling approaches further support validation by comparing predictions with independent datasets or biological observations across scales [77,78]. These examples illustrate emerging efforts toward more robust validation beyond internal consistency checks.

Transparency is generally adequate at the methodological level. Most studies clearly describe modeling assumptions, system setup, and force field choices, allowing readers to understand how NP–membrane interactions are represented. However, external validation using independent datasets remains rare, and uncertainty quantification is seldom reported in current NP–BBB modeling studies, which limits confidence in predictive generalizability.

## Discussion

This section synthesizes the main findings from our systematic review of 56 computational studies on NP transport across the BBB. It also reflects on limitations of the review process and outlines directions for future work toward more integrated and predictive modeling frameworks.

### Implications

#### Modeling landscape

The reviewed studies show that current in silico approaches offer complementary strengths across modeling scales. Mechanistic simulations and data-driven models provide distinct yet related insights into protein corona formation, NP–membrane interactions, and cross-BBB transport. These approaches address different stages of the transport process, although opportunities remain to develop more unified and predictive models of NP–BBB permeability. Our taxonomy clarifies 5 methodological domains: molecular simulations, descriptor-based QSAR/QSPR models, data-driven AI approaches, system-level PK/PD and PBPK frameworks, and nanoinformatics infrastructures.

A cross-scale comparison of current in silico studies shows that modeling choices depend strongly on how NPs and BBB components are represented across molecular, cellular, and system levels. Rather than relying on visual or purely geometric models, these approaches encode NPs and their biological transport environments through structured numerical, categorical, and vectorized features that enable quantitative computation. The reviewed literature summarizes 5 complementary descriptor classes: conventional NP physicochemical properties, computational and topological encodings, protein corona fingerprints, simulation-derived mechanistic and transport parameters, and system-level pharmacokinetic variables that reveal permeability or transport efficiency.

#### Predictive workflows

The analysis of model performance and validation strategies reveals several consistent trends. Data-driven models benefit from careful preprocessing and standardization. Across studies, corona-derived descriptors often outperform intrinsic physicochemical properties in predicting NP association or uptake. Robust validation practices such as stratified sampling, cross-validation, external test sets, and experimental confirmation using mass spectrometry or imaging improve confidence in model predictions.

There is growing recognition of the need for integrated computational–experimental workflows to improve predictive reliability and support rational NP design for BBB transport. Molecular simulations, experimental assays, and ML models are still often used separately, which limits their combined predictive power. Iterative pipelines are emerging in which mechanistic simulations generate interpretable descriptors, experimental platforms provide validation data, and ML models integrate these inputs to guide candidate selection.

#### Multilevel challenges

Many models still focus on isolated stages of NP transport. They often rely on small or imbalanced datasets and use heterogeneous experimental protocols. This makes cross-study comparison difficult and limits predictive integration. These observations point to the need for BBB-specific datasets and harmonized experimental standards, as well as closer integration across molecular, cellular, and system-level models.

**Fig. 6.**
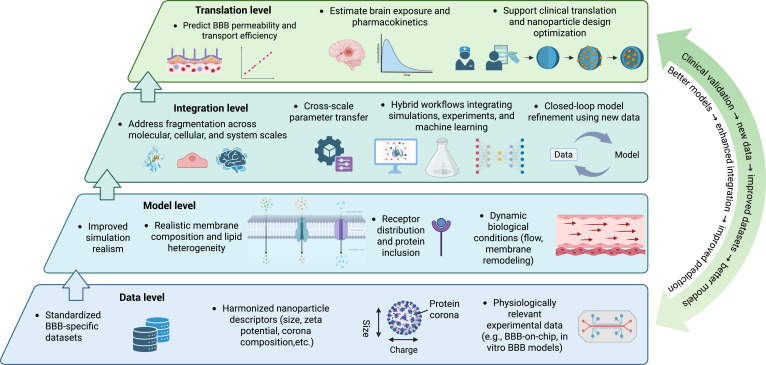
Multilevel challenges and future directions for improving in silico modeling of nanoparticle transport across the BBB. High-quality, standardized datasets (data level) provide the foundation for building physiologically realistic models (model level). Improved model realism supports cross-scale integration and hybrid computational–experimental workflows (integration level), which are essential for bridging the gap between in silico predictions and clinical translation (translation level). Continuous feedback between these levels enables iterative refinement of predictive frameworks.

Progress in in silico modeling of NP transport across the BBB depends on advances across several interconnected levels rather than improvements in a single domain. Current limitations relate to data quality, model realism, cross-scale integration, and clinical translation. These levels are interdependent, as shown in Fig. 6. Improvements at the data and model levels can support better integration, while stronger integration may improve translational relevance. Addressing these multilevel challenges is important for moving from fragmented tools toward predictive frameworks.

##### Data-level challenges

High-quality datasets that directly quantify BBB-specific permeability remain limited. Many studies rely on proxy measurements from non-BBB systems or heterogeneous experimental conditions. This reduces physiological relevance and complicates cross-study comparison. Descriptor reporting is also inconsistent. Parameters such as size, surface chemistry, and corona composition are often measured under different protocols. The lack of harmonized standards makes it difficult to combine datasets or build robust predictive models. Limited access to raw data, descriptor tables, and preprocessing workflows further restricts reproducibility. Wider adoption of FAIR data practices and standardized reporting may support more reliable model development.

##### Model-level challenges

Many computational studies employ simplified membrane representations when modeling NP–BBB interactions. Symmetric lipid bilayers with limited compositional diversity are commonly used. Key structural features of the BBB, such as lipid heterogeneity, asymmetric leaflet composition, and membrane proteins or transport receptors, are often simplified or omitted. Dynamic physiological conditions, including membrane remodeling or flow-related effects, are also rarely incorporated. In addition, most studies apply established simulation methods such as AAMD or CGMD rather than introducing new methodological approaches. Advances are often driven by how these methods are applied to BBB-relevant systems, rather than by algorithmic innovation. These modeling choices influence how NP–BBB interactions are represented and interpreted across studies.

##### Integration-level challenges

Current in silico approaches are often developed at separate scales, such as molecular simulations, data-driven models, and system-level frameworks. Many studies focus on a single stage or modeling scale, and explicit linkage between these approaches remains limited. Possible coupling approaches, such as hierarchical parameter transfer or hybrid multiscale frameworks, may help connect models across scales, although their practical implementation remains limited. ML models are frequently applied as stand-alone predictors, rather than as part of integrated modeling workflows. Hybrid simulation–experiment–AI pipelines and unified nanoinformatics infrastructures are still emerging. This separation influences how mechanistic insights, predictive models, and system-level outcomes are connected when interpreting BBB transport.

##### Translation-level challenges

Despite methodological progress, translating computational predictions into clinically relevant insights remains challenging. Many reviewed studies rely on proxy endpoints such as cellular uptake rather than BBB-specific permeability metrics (e.g., Papp or Pe). This limits their direct relevance to brain exposure outcomes. External validation using BBB-relevant datasets is also uncommon, and only a small subset of studies attempts cross-scale linkage to PK/PBPK models to support system-level predictions of brain exposure. As a result, alignment between in silico predictions and clinically meaningful transport efficiency remains limited. This suggests the need for iterative integration of human-relevant validation systems and cross-scale modeling.

### Limitations

This review is limited to published, English-language literature and relies on predefined inclusion criteria that may introduce selection bias. Comparing highly heterogeneous modeling methodologies also poses inherent challenges. To mitigate these issues, we applied transparent inclusion/exclusion criteria, used a standardized coding template, performed double-checking of assignments, and provide full study lists and category mappings in the Supplementary Materials for reproducibility. Despite these limitations, the analysis robustly demonstrates the complementary strengths of the 5 computational domains and the transformative potential of their integration for brain drug delivery.

### Future work

#### Data foundations and reproducibility

Future progress in NP–BBB modeling may benefit from curated BBB-specific datasets supported by harmonized experimental protocols and broader adoption of FAIR data principles. Many current studies rely on heterogeneous experimental conditions or proxy cell systems, which can limit physiological relevance and cross-study benchmarking. Limited dataset diversity may also introduce material bias, increasing the risk of overfitting and reducing model transferability across nanomaterial types. Public deposition of datasets and code, together with transparent documentation of preprocessing workflows and model parameters, can improve reproducibility and enable cross-study comparison. Independent validation datasets, cross-study benchmarking, systematic sensitivity analyses, and transparent uncertainty reporting may further strengthen predictive reliability.

#### Cross-scale modeling and biological realism

Future efforts should focus on connecting molecular simulations, experimental platforms, and system-level PK/PD or PBPK models to better represent the full NP transport process across the BBB. Linking mechanistic simulations with predictive models and exposure analyses can enable more comprehensive descriptions of the transport sequence, from protein adsorption and membrane interaction to brain distribution. Upscaling approaches, such as population balance equations or agent-based modeling, may help relate heterogeneous single-cell behavior to barrier-level transport outcomes. Improvements in simulation realism, including heterogeneous lipid composition, receptor dynamics, tight junction structures, flow-related conditions, and additional biological features such as the endothelial glycocalyx and multicellular interactions within the NVU, may support more physiologically meaningful predictions. Future models may also benefit from incorporating regional BBB heterogeneity and disease-altered barrier states (e.g., in glioblastoma or neurodegenerative conditions).

#### AI-integrated nanoinformatics workflows

Nanoinformatics provides a foundation for integrating experimental datasets, simulation outputs, and predictive models within unified computational pipelines. Future work may benefit from prioritizing standardized databases that link NP descriptors with well-annotated experimental metadata under harmonized conditions. Advanced AI approaches, including causal and hybrid simulation–AI methods, may support more interpretable modeling and iterative design strategies. Emerging scientific ML approaches, which embed physical or biological constraints into learning frameworks, may further help bridge the gap between predictive performance and mechanistic understanding.

**Fig. 7.**
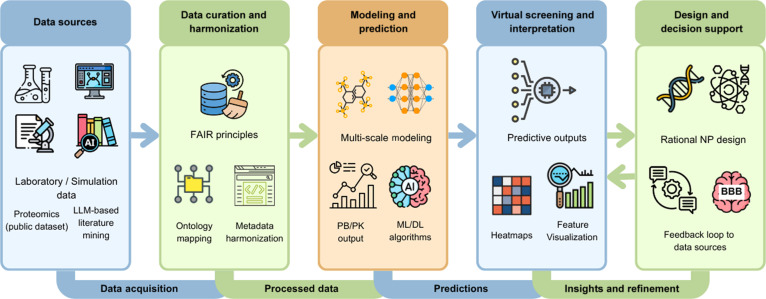
Overview of the future artificial intelligence (AI)-integrated nanoinformatics workflow. The process starts with AI-automated data curation to build FAIR (Findable, Accessible, Interoperable, and Reusable) datasets for predictive modeling. These models guide the synthesis of specific nanoparticle designs for testing on BBB-on-chip platforms. The experimental results then feed back into the system, enabling dynamic decision-making and continuous optimization of future designs. This workflow illustrates a shift from static, analysis-oriented modeling toward a closed-loop, AI-driven design paradigm for BBB-targeted nanomedicine.

Building upon this foundation, the workflow shown in Fig. 7 illustrates a lab-in-the-loop paradigm. In this iterative cycle, computational models generate predictions that guide candidate selection for BBB-on-chip testing. Experimental outputs are then fed back into the modeling framework to refine datasets, recalibrate simulations, and retrain predictive models. Such closed-loop workflows can improve model generalizability and support more transparent NP design.

Open-access nanoinformatics platforms may further support benchmarking, collaborative validation, and systematic integration of data and models.

## Conclusion

This review mapped current in silico methods for modeling NP transport across the BBB, covering both physics-based simulations and data-driven approaches. We established a methodological taxonomy and clarified how NPs and BBB components are represented, as well as how modeling scales and applications are used across molecular, cellular, and system levels. Our assessment of data quality and validation practices highlights the need for standardized descriptors and reproducible workflows. By presenting strategies to integrate multiscale information into a unified workflow, this study links data collection, modeling, and design. This framework is expected to accelerate the rational, transparent, and nonanimal-reliant design of brain-targeted nanomedicines, with potential to reduce attrition in CNS drug development, support regulatory decision-making, and complement or partially replace animal studies.

## Supplementary Materials

Supplementary 1 Supplementary Notes Tables S1 to S5
