# Supplementary material for: In Silico Modeling of Nanoparticle Transport across the Blood–Brain Barrier: A Systematic Review
Source: Comput Struct Biotechnol J. 2026 Mar 25;35(3):0020. doi: 10.34133/csbj.0020 (PMC13394976; doi:10.34133/csbj.0020)
Supplement: Supplementary 1 — Supplementary Notes Tables S1 to S5 [file csbj.0020.f1.zip › ReviewPaper_Supplementary_material.pdf]

In Silico Modeling of Nanoparticle Transport Across the Blood–Brain Barrier: A Systematic Review

Supplementary Materials

SM1: METHODS - SEARCH STRATEGY AND KEY TERMS

The key terms are grouped into three thematic components. Within each component, the keywords listed in Table S1 are combined using the OR Boolean operator. The overall systematic search was performed with the combined query string **P1 AND P2 AND P3**. For the search implementation, stricter constraints were applied to ensure precision: terms related to the blood–brain barrier and computational modeling (**P2** and **P3**) were searched only within the *title field*, while nanoparticle-related terms (**P1**), due to their broader variability and numerous synonyms, were searched within both the *title and abstract fields*. All searches were limited to *English-language journal articles* published up to May 2025 and results are shown in Table S2.

Table S1. Key terms used in the search, grouped into the three thematic components (P1–P3).

Component	Search Terms (Boolean OR within each component)
(P1) Nanoparticle Context	"nanoparticle*" OR "nanocarrier*" OR "nanodrug*" OR "nano-drug*" OR "nanomedicine*" OR "nanomedicines" OR "nanoparticle drug delivery" OR "nano-based drug delivery" OR "nanodelivery system*" OR "nanochannel*" OR "spherical nucleic acid*" OR SNA OR "gold nanoparticle*" OR "mesoporous silica nanoparticle*" OR MSN OR "metal-organic framework*" OR "metal organic framework*" OR MOF OR "polymeric micelle*" OR "micellar nanoparticle*" OR "lipid nanoparticle*" OR LNP OR "extracellular vesicle*" OR EV OR "exosome*".
(P2) Blood–Brain Barrier and Transport Mechanisms	"blood-brain barrier" OR "brain blood barrier" OR BBB OR "blood-brain barrier permeability" OR "BBB permeability" OR "blood-brain barrier penetration" OR "BBB penetration" OR "blood-brain barrier transport" OR "BBB transport" OR "blood-brain barrier permeation" OR "BBB permeation" OR "across the blood-brain barrier" OR "across the BBB" OR "brain delivery" OR "brain targeting" OR "brain uptake" OR "brain accumulation" OR "brain distribution" OR "central nervous system" OR CNS OR "brain endothelial cells" OR "nanoparticle uptake" OR "cellular uptake" OR "cellular internalization" OR "cellular interaction" OR "nanoparticle-cell interaction" OR "NP-cell interaction" OR "membrane interaction" OR "membrane adhesion" OR "membrane translocation" OR "membrane fusion" OR "nanoparticle-membrane interaction" OR "transcytosis" OR "adsorptive-mediated transcytosis" OR AMT OR "receptor-mediated transcytosis" OR RMT OR "carrier-mediated transport" OR CMT OR "endocytosis" OR "passive diffusion" OR "passive transport" OR "active efflux" OR "ABC-efflux pump*" OR "P-glycoprotein" OR P-gp OR "protein corona" OR "biomolecular corona" OR "corona formation" OR "corona composition" OR "corona proteins" OR "protein adsorption" OR "plasma protein adsorption" OR "electrostatics" OR "electrostatics modelling".
(P3) In Silico and Computational Methods	"in silico" OR "in silico modeling" OR "in silico simulation" OR "computational modeling" OR "computational model*" OR "computational method*" OR "computational approach*" OR "computational simulation*" OR "molecular modeling" OR "molecular dynamics" OR "coarse-grained molecular dynamics" OR CGMD OR "dissipative particle dynamics" OR DPD OR "molecular docking" OR "virtual screening" OR "pharmacokinetic modeling" OR "AI-driven" OR "AI-assisted" OR "machine learning" OR "deep learning" OR "artificial intelligence" OR AI OR "prediction model*" OR "predictive model*" OR "predictive modeling" OR "predictive platform" OR "property prediction" OR prediction OR classification OR "predict*" OR "quantitative structure-activity relationship*" OR QSAR OR "quantitative structure-property relationship*" OR QSPR OR "data-driven model*" OR "mechanistic model*" OR "statistical model*" OR "statistical learning" OR "statistical analysis" OR modelling OR modeling OR simulation OR "correlation*".

Table S2. Summary of database search queries and number of records retrieved.

No.	Database	Search Query (Simplified Expression)	Papers Found
1	PubMed	([**P1**:Title/Abstract]) **AND** ([**P2**:Title]) **AND** ([**P3**:Title])	119
2	Scopus	(TITLE-ABS-KEY(**P1**)) **AND** (TITLE(**P2**)) **AND** (TITLE(**P3**))	145
3	Web of Science	TS=(**P1**) **AND** TI=(**P2**) **AND** TI=(**P3**)	407

SM2: INCLUDED STUDIES AND THEIR MODELING CATEGORIES

Table S3 lists the 56 studies included in this systematic review, organized by their main computational modeling direction. The studies are grouped into two major categories identified during

data extraction:**Physics-Based Molecular Simulation Approaches** and **Data-Driven Modeling**. Each entry provides the modeling sub-approach, title and study reference used in the main manuscript.

Table S3. **Summary of included studies categorized by sub-approach.**

No.	Sub-Approach	Study	Title / Reference
**Physics-Based Molecular Simulation Approaches (32 studies)**			
1	All-Atom Molecular Dynamics (AAMD)	Lee (2025)	Molecular Dynamics Simulations of Protein Corona Formation on Membrane Surfaces: Effects of Lipid Composition and PEGylation on Selective Plasma Protein Adsorption
2	All-Atom Molecular Dynamics (AAMD)	Bini et al. (2023)	Deconstructing Electrostatics of Functionalized Metal Nanoparticles from Molecular Dynamics Simulations
3	All-Atom Molecular Dynamics (AAMD)	Arcangeli et al. (2019)	Characterization of blood–brain barrier crossing and tumor homing peptides by molecular dynamics simulations
4	All-Atom Molecular Dynamics (AAMD)	Mousavi and Hashemianzadeh (2018)	Poly(*n*-butyl cyanoacrylate) as a nanocarrier for rivastigmine transport across the blood–brain barrier in Alzheimer's disease treatment: a perspective from molecular dynamics simulations
5	Coarse-Grained Molecular Dynamics (CGMD)	Chen et al. (2023)	Mesoscopic simulations of protein corona formation on zwitterionic peptide-grafted gold nanoparticles
6	Coarse-Grained Molecular Dynamics (CGMD)	Li et al. (2023)	The effects of serum albumin pre-adsorption of nanoparticles on protein corona and membrane interaction: A molecular simulation study
7	Coarse-Grained Molecular Dynamics (CGMD)	Azman et al. (2021)	Dynamics of Human Serum Albumin Corona Formation on Gold Nanorods with Different Surface Ligands *in silico*
8	Coarse-Grained Molecular Dynamics (CGMD)	Shen et al. (2018)	Understanding receptor-mediated endocytosis of elastic nanoparticles through coarse-grained molecular dynamics simulation
9	Coarse-Grained Molecular Dynamics (CGMD)	Shao and Hall (2016)	Protein adsorption on nanoparticles: Model development using computer simulation
10	Dissipative Particle Dynamics (DPD)	Sun et al. (2023)	Tuning the Microstructure of Protein Corona by Nanoparticle Hydrophobicity: A Dissipative Particle Dynamics Study

Continued on next page.

(Continued) Summary of included studies categorized by sub-approach.

No.	Sub-Approach	Study	Title / Reference
11	Dissipative Particle Dynamics (DPD)	Li et al. (2022)	*In silico* insights into the receptor-mediated endocytosis of virus-like nanoparticles
12	Dissipative Particle Dynamics (DPD)	Xia et al. (2018)	Design strategy of pH-sensitive triblock copolymer micelles for efficient cellular uptake by computer simulations
13	Dissipative Particle Dynamics (DPD)	Li and Gorfe (2015)	Receptor-mediated membrane adhesion of lipid-polymer hybrid (LPH) nanoparticles studied by dissipative particle dynamics simulations
14	Dissipative Particle Dynamics (DPD)	Mao et al. (2014)	Simulation and analysis of cellular internalization pathways and membrane perturbation for graphene nanosheets
15	Dissipative Particle Dynamics (DPD)	Ding and Ma (2013)	Design maps for cellular uptake of gene nanovectors by computer simulation
16	Dissipative Particle Dynamics (DPD)	Li et al. (2012)	Molecular modeling of the relationship between nanoparticle shape anisotropy and endocytosis kinetics
17	Dissipative Particle Dynamics (DPD)	Li et al. (2017)	Counterintuitive cooperative endocytosis of like-charged nanoparticles in cellular internalization: Computer simulation and experiment
18	Dissipative Particle Dynamics (DPD)	Ding and Ma (2014)	Computer simulation of the role of protein corona in cellular delivery of nanoparticles
19	Multiscale Simulation	Subbotina et al. (2025)	UANanoDock: A Web-Based UnitedAtom Multiscale Nanodocking Tool for Predicting Protein Adsorption onto Nanoparticles
20	Multiscale Simulation	Schneemilch and Quirke (2022)	Predicting nanoparticle uptake by biological membranes: theory and simulation
21	Multiscale Simulation	Sarker et al. (2022)	Multiscale Simulation of Protein Corona Formation on Silver Nanoparticles: Study of Ovispirin-1 Peptide Adsorption
22	Multiscale Simulation	Power et al. (2019)	A multiscale model of protein adsorption on a nanoparticle surface

Continued on next page.

(Continued) Summary of included studies categorized by sub-approach.

No.	Sub-Approach	Study	Title / Reference
23	Multiscale Simulation	Tavanti et al. (2019)	Multiscale Molecular Dynamics Simulation of Multiple Protein Adsorption on Gold Nanoparticles
24	Multiscale Simulation	Sajib et al. (2020)	Protein corona on gold nanoparticles studied with coarse-grained simulations
25	Hybrid / Enhanced Sampling	Penna et al. (2020)	Nanoscale: *In silico* classification of ligand functionalised surfaces for protein adsorption resistance
26	Hybrid / Enhanced Sampling	Lunnoo et al. (2019)	*In silico* study of gold nanoparticle uptake into a mammalian cell: interplay of size, shape, surface charge, and aggregation
27	Hybrid / Enhanced Sampling	Delle Piane et al. (2018)	Molecular dynamics simulations of the silica-cell membrane interaction: insights on biomineralization and nanotoxicity
28	Hybrid / Enhanced Sampling	Quan et al. (2017)	Understanding the cellular uptake of pH-responsive zwitterionic gold nanoparticles: a computer simulation study
29	Hybrid / Enhanced Sampling	Hong et al. (2017)	A hybrid molecular dynamics / multiconformer continuum electrostatics (MD/MCCE) approach for the determination of surface charge of nanomaterials
30	Hybrid / Enhanced Sampling	Pedram et al. (2016)	Optimal magnetic field for crossing super-paramagnetic nanoparticles through the brain blood barrier: a computational approach
31	Hybrid / Enhanced Sampling	Shamloo et al. (2016)	Computing the blood–brain barrier (BBB) diffusion coefficient: a molecular dynamics approach
32	Hybrid / Enhanced Sampling	Pedram et al. (2014)	Modeling and simulation of crossing magnetic nanoparticles through the blood–brain barrier (BBB)
**Data-Driven Modeling (24 studies)**			
33	QSAR / QSPR Modeling	Walkey et al. (2014)	Protein corona fingerprinting predicts the cellular interaction of gold and silver nanoparticles

Continued on next page.

(Continued) Summary of included studies categorized by sub-approach.

No.	Sub-Approach	Study	Title / Reference
34	QSAR / QSPR Modeling	Liu et al. (2015)	Prediction of nanoparticles–cell association based on corona proteins and physicochemical properties
35	QSAR / QSPR Modeling	Palchetti et al. (2016)	Nanoparticles–cell association predicted by protein corona fingerprints
36	QSAR / QSPR Modeling	Bigdeli et al. (2016)	Exploring cellular interactions of liposomes using protein corona fingerprints and physicochemical properties
37	QSAR / QSPR Modeling	Sengottiyan et al. (2023)	Core, coating, or corona? The importance of considering protein coronas in nano-QSPR modeling of zeta potential
38	Machine Learning & Deep Learning	Findlay et al. (2018)	Machine learning provides predictive analysis into silver nanoparticle protein corona formation from physicochemical properties
39	Machine Learning & Deep Learning	Duan et al. (2020)	Prediction of protein corona on nanomaterials by machine learning using novel descriptors
40	Machine Learning & Deep Learning	Ban et al. (2020)	Machine learning predicts the functional composition of the protein corona and the cellular recognition of nanoparticles
41	Machine Learning & Deep Learning	Ouassil et al. (2022)	Supervised learning model predicts protein adsorption to carbon nanotubes
42	Machine Learning & Deep Learning	Fu et al. (2024)	Machine learning enables comprehensive prediction of the relative protein abundance of multiple proteins on the protein corona
43	Machine Learning & Deep Learning	Liao et al. (2024)	Unveiling protein corona composition: predicting with resampling embedding and machine learning
44	Machine Learning & Deep Learning	Cenk et al. (2014)	Artificial neural network modeling and simulation of *in vitro* nanoparticle–cell interactions
45	Machine Learning & Deep Learning	Bilgi et al. (2024)	Identifying factors controlling cellular uptake of gold nanoparticles by machine learning

Continued on next page.

(Continued) Summary of included studies categorized by sub-approach.

No.	Sub-Approach	Study	Title / Reference
46	Machine Learning & Deep Learning	Rafieioskouei et al. (2025)	Beyond correlation: establishing causality in protein corona formation for nanomedicine
47	Machine Learning & Deep Learning	Iaquinta et al. (2024)	Machine learning for the sensitivity analysis of a model of the cellular uptake of nanoparticles for the treatment of cancer
48	Machine Learning & Deep Learning	Papa et al. (2016)	Investigation of the influence of protein corona composition on gold nanoparticle bioactivity using machine learning approaches
49	Machine Learning & Deep Learning	Yang et al. (2023)	Following nanoparticle uptake by cells using high-throughput microscopy and the deep-learning-based cell identification algorithm Cellpose
50	Pharmacokinetic / Pharmacodynamic (PK/PD) Modeling	Riviere et al. (2018)	Modeling gold nanoparticle biodistribution after arterial infusion into perfused tissue
51	Pharmacokinetic / Pharmacodynamic (PK/PD) Modeling	Hu et al. (2019)	Understanding the influence of nanocarrier-mediated brain delivery on therapeutic performance through PK/PD modeling
52	Pharmacokinetic / Pharmacodynamic (PK/PD) Modeling	Kadakia et al. (2019)	Mathematical modeling and simulation to investigate the CNS transport characteristics of nanoemulsions
53	Pharmacokinetic / Pharmacodynamic (PK/PD) Modeling	Deng et al. (2019)	Endocytosis mechanism in physiologically based pharmacokinetic modeling of nanoparticles
54	Pharmacokinetic / Pharmacodynamic (PK/PD) Modeling	Abd-algaleel et al. (2021)	Synchronizing *in silico*, *in vitro*, and *in vivo* studies for the successful nose-to-brain delivery of an anticancer molecule

Continued on next page.

(Continued) Summary of included studies categorized by sub-approach.

No.	Sub-Approach	Study	Title / Reference
55	Nanoinformatics Framework	Afantitis et al. (2018)	A nanoinformatics decision support tool for the virtual screening of gold nanoparticle cellular association using protein corona fingerprints
56	Nanoinformatics Framework	Hasenkopf et al. (2022)	Computational prediction and experimental analysis of the nanoparticle–protein corona: showcasing an *in vitro–in silico* workflow providing FAIR data

SM3: SCORING RUBRIC AND BIAS DIMENSIONS

To ensure methodological rigor and consistency, each included study was evaluated according to the three assessment criteria summarized in Table S4. The scoring rubric below defines the 0–2 scale applied to each criterion, corresponding respectively to low, moderate, and high levels of methodological quality and reproducibility. All studies were scored independently by two reviewers, and the average score was used to determine the overall quality level (Table S5).

Table S4. **Scoring rubric for AC1–AC3 evaluation.**

Criterion (AC)	Score Range	Scoring Description
**AC1: Model Simplification Bias**	0–2	0 = **Oversimplified / Irrelevant:** Models only basic NP–biomolecule interactions in bulk solution (Phase 1) without any representation of cellular or membrane-level processes; no transport mechanism included.<br><br>1 = **Partial General Stage:** Represents one key transport stage (Phase 1, 2, or 3) using a generic or non-BBB-specific environment (e.g., universal lipid bilayer, general mammalian cell membrane, or plasma incubation with inferred cellular association).<br><br>2 = **High Fidelity / Multi-Stage:** Models multiple BBB transport stages, *or* explicitly includes BBB-representative features such as endothelial cells, tight junctions, P-gp transporters, or receptor-mediated transcytosis mechanisms.<br><br>*Note:* Classification follows the three-stage BBB transport framework: (1) Protein corona formation; (2) NP–membrane interaction; (3) Cross-BBB passage (endocytosis / transcytosis).
**AC2: Data Availability Bias**	0–2	0 = **Insufficient / Non-reproducible:** Data, model inputs, or computational details are incomplete or missing, preventing reproducibility or independent verification. Neither simulation inputs (e.g., force fields, configuration files, trajectories) nor datasets or model details are adequately documented or shared.<br><br>1 = **Transparent but Not Open:** Methodology, parameters, and model setup are clearly and transparently described, allowing conceptual reproducibility, but the underlying datasets, code, or configuration files are not publicly accessible.<br><br>2 = **Transparent and Open:** Builds upon transparent and well-documented reporting (as in score 1) and additionally provides openly accessible data, input files, scripts, or repositories following open-science and FAIR principles, ensuring full reproducibility and reuse.
**AC3: Validation Bias**	0–2	0 = **No or Unclear Validation:** The study does not include any explicit validation procedure, or the origin of the data and benchmarking criteria are unclear. There is no comparison with experiments, literature references, or independent datasets.<br><br>1 = **Partial or Qualitative Validation:** The model shows qualitative consistency or limited benchmarking, such as comparison with trends reported in previous studies or simplified experimental data. However, the validation lacks quantitative rigor, comprehensive metrics, or methodological clarity (e.g., missing uncertainty estimates or test-set details).<br><br>2 = **Comprehensive and Transparent Validation:** The study provides systematic and reproducible validation. For simulation studies, results are compared quantitatively or qualitatively with experimental data, literature benchmarks, or multi-scale simulations to ensure physical consistency. For data-driven and machine learning models, validation includes cross-validation, independent or external test datasets, and reporting of performance metrics (e.g., R^2, RMSE, accuracy), with any wet-lab or experimental confirmation supporting model predictivity and generalizability.

SM4: AC1–AC3 QUALITY ASSESSMENT TABLE

Table S5 summarizes the methodological quality evaluation of all 56 included studies according to the three assessment criteria (AC1–AC3) defined in the main text and detailed in Section SM4.

Table S5. **Summary of AC1–AC3 quality assessment for representative studies**

No.	Study (First Author, Year)	AC1	AC2	AC3	Average Score
1	Lee (2025)	1	1	1	1.00
2	Bini et al. (2023)	0	2	1	1.00
3	Arcangeli et al. (2019)	1	1	1	1.00
4	Mousavi and Hashemianzadeh (2018)	1	1	1	1.00
5	Chen et al. (2023)	1	2	1	1.33
6	Li et al. (2023)	1	1	2	1.33
7	Azman et al. (2021)	1	1	1	1.00
8	Shen et al. (2018)	1	1	2	1.33
9	Shao and Hall (2016)	1	2	1	1.33
10	Sun et al. (2023)	0	1	1	0.67
11	Li et al. (2022)	1	1	1	1.00
12	Xia et al. (2018)	1	1	1	1.00
13	Li and Gorfe (2015)	1	1	1	1.00
14	Mao et al. (2014)	1	1	1	1.00
15	Ding and Ma (2013)	1	1	1	1.00
16	Li et al. (2012)	1	1	1	1.00
17	Li et al. (2017)	1	1	1	1.00
18	Ding and Ma (2014)	1	1	1	1.00
19	Subbotina et al. (2025)	1	2	2	1.67
20	Schneemilch and Quirke (2022)	1	2	2	1.67
21	Sarker et al. (2022)	1	2	1	1.33
22	Power et al. (2019)	1	2	1	1.33
23	Tavanti et al. (2019)	1	1	2	1.33
24	Jahan Sajib et al. (2020)	1	2	2	1.67
25	Penna and Yarovsky (2020)	1	2	1	1.33
26	Lunnoo et al. (2019)	1	2	2	1.67
27	Delle Piane et al. (2018)	1	2	2	1.67
28	Quan et al. (2017)	2	2	2	2.00
29	Hong et al. (2017)	1	2	2	1.67
30	Pedram et al. (2016)	2	1	1	1.33
31	Shamloo et al. (2016)	2	1	1	1.33
32	Pedram et al. (2014)	2	1	1	1.33
33	Walkey et al. (2014)	1	2	2	1.67
34	Liu et al. (2015)	1	2	2	1.67
35	Palchetti et al. (2016)	1	2	1	1.33
36	Bigdeli et al. (2016)	1	1	2	1.33
37	Sengottiyan et al. (2023)	0	2	2	1.33
38	Findlay et al. (2018)	1	2	2	1.67
39	Duan et al. (2020)	1	2	2	1.67
40	Ban et al. (2020)	1	2	2	1.67
41	Ouassil et al. (2022)	1	2	2	1.67
42	Fu et al. (2024)	1	2	2	1.67
43	Liao et al. (2024)	1	2	2	1.67
44	Cenk et al. (2014)	1	1	1	1.00
45	Bilgi et al. (2024)	1	2	2	1.67
46	Rafieioskouei et al. (2025)	1	2	1	1.33
47	Iaquinta et al. (2024)	1	2	1	1.33
48	Papa et al. (2016)	1	2	2	1.67
49	Yang et al. (2023)	1	2	2	1.67
50	Riviere et al. (2018)	1	1	1	1.00
51	Hu et al. (2019)	2	1	1	1.33
52	Kadakia et al. (2019)	2	1	2	1.67
53	Deng et al. (2019)	1	1	1	1.00
54	Abd-algaleel et al. (2021)	2	1	2	1.67
55	Afantitis et al. (2018)	1	2	2	1.67
56	Hasenkopf et al. (2022)	1	2	2	1.67

Scores: 0 = absent, 1 = partial, 2 = comprehensive. The complete table of 56 studies is provided in the supplementary dataset (Excel file).
